# Proteomic analysis reveals ginsenoside Rb1 attenuates myocardial ischemia/reperfusion injury through inhibiting ROS production from mitochondrial complex I

**DOI:** 10.7150/thno.43895

**Published:** 2021-01-01

**Authors:** Lujing Jiang, Xiaojian Yin, Ya-Hui Chen, Yan Chen, Wei Jiang, Hao Zheng, Feng-Qing Huang, Baolin Liu, Wei Zhou, Lian-Wen Qi, Jia Li

**Affiliations:** 1 State Key Laboratory of Natural Medicines, School of Traditional Chinese Pharmacy, China Pharmaceutical University, Nanjing 210009, Jiangsu, China.; 2 Clinical Metabolomics Center, China Pharmaceutical University, Nanjing 211198, Jiangsu, China.

**Keywords:** Ginsenoside Rb1, Mitochondrial complex I, Myocardial ischemia/reperfusion injury, Proteomic analysis, Reactive oxygen species

## Abstract

**Rationale:** Reactive oxygen species (ROS) burst from mitochondrial complex I is considered the critical cause of ischemia/reperfusion (I/R) injury. Ginsenoside Rb1 has been reported to protect the heart against I/R injury; however, the underlying mechanism remains unclear. This work aimed to investigate if ginsenoside Rb1 attenuates cardiac I/R injury by inhibiting ROS production from mitochondrial complex I.

**Methods:** In *in vivo* experiments, mice were given ginsenoside Rb1 and then subjected to I/R injury. Mitochondrial ROS levels in the heart were determined using the mitochondrial-targeted probe MitoB. Mitochondrial proteins were used for TMT-based quantitative proteomic analysis. In *in vitro* experiments, adult mouse cardiomyocytes were pretreated with ginsenoside Rb1 and then subjected to hypoxia and reoxygenation insult. Mitochondrial ROS, NADH dehydrogenase activity, and conformational changes of mitochondrial complex I were analyzed.

**Results:** Ginsenoside Rb1 decreased mitochondrial ROS production, reduced myocardial infarct size, preserved cardiac function, and limited cardiac fibrosis. Proteomic analysis showed that subunits of NADH dehydrogenase in mitochondrial complex I might be the effector proteins regulated by ginsenoside Rb1. Ginsenoside Rb1 inhibited complex I- but not complex II- or IV-dependent O_2_ consumption and enzyme activity. The inhibitory effects of ginsenoside Rb1 on mitochondrial I-dependent respiration and reperfusion-induced ROS production were rescued by bypassing complex I using yeast NADH dehydrogenase. Molecular docking and surface plasmon resonance experiments indicated that ginsenoside Rb1 reduced NADH dehydrogenase activity, probably via binding to the ND3 subunit to trap mitochondrial complex I in a deactive form upon reperfusion.

**Conclusion:** Inhibition of mitochondrial complex I-mediated ROS burst elucidated the probable underlying mechanism of ginsenoside Rb1 in alleviating cardiac I/R injury.

## Introduction

Acute myocardial infarction remains a major cause of death and disability worldwide. The obvious remedy for ischemia is to restore blood flow as quickly as possible; but, restoration of oxygenated blood can induce further damage beyond that of the initial ischemic injury, which is known as ischemia/reperfusion (I/R) injury 1, 2. Though I/R injury impairs cardiac function via diverse mechanisms 3-5, a recent study indicates that a burst of reactive oxygen species (ROS) from the mitochondrial respiratory chain initiates a cascade of events that result in tissue damage 6. ROS directly damage DNA and, together with mitochondrial Ca^2+^ influx, impair mitochondrial function via prolonged opening of mitochondrial permeability transition pore (MPTP) 7. Therefore, limiting ROS production upon reperfusion appears to be an appealing therapeutic strategy to diminish I/R injury. However, the results from both experimental and clinical studies of antioxidants are mixed and mostly negative 2, 8. A probable reason could be that the precise underlying molecular mechanisms for ROS production remain elusive.

The mitochondrial electron transport chain is the most important source of ROS generation 9. It has been demonstrated that the citric acid cycle intermediate succinate builds up dramatically in cardiomyocytes during ischemia. Upon reperfusion, the accumulated succinate serves as a reducing equivalent, driving superoxide production at mitochondrial complex I through reverse electron transfer (RET) reaction (*i.e.*, electron transfer from reduced ubiquinone to NAD^+^) 10, 11. Because of extensive production of superoxide at mitochondrial complex I, blockade of electron transport at this point might decrease RET-mediated ROS production and protect the heart from I/R injury. In this regard, rotenone has been shown to reduce ROS production upon reperfusion by inhibiting mitochondrial complex I 12. However, rotenone irreversibly inhibits mitochondrial complex I, thereby limiting its potential application in preventing ischemic injury 13.

The potential site of mitochondrial complex I that drives ROS production is flavin mononucleotide (FMN) in NADH dehydrogenase 14. During reperfusion, a highly reduced ubiquinone, in conjunction with a high membrane potential and a low rate of ATP synthesis, drives electrons from the reduced coenzyme Q (CoQ) pool back to NADH dehydrogenase in complex I, wherein the reduced FMN reacts with oxygen to generate superoxide 10, 14. The flavin inhibitor diphenyleneiodonium blocks RET-mediated ROS production from complex I 15. Moreover, mitochondrial fission inhibitor mdivi-1 has been found to decrease ROS production from RET via inhibition of NADH dehydrogenase activity 16. Mitochondrial complex I undergoes a conformational change based on the availability of oxygen. Limited oxygen supply during ischemia shifts mitochondrial complex I from the normal active form to a deactive state 17. The deactive form is characterized by a loss of catalytic activity to generate ROS 18. Thus, transient trapping of complex I in the deactive form upon reperfusion may be a potential therapeutic strategy to limit RET-driven ROS generation. These events throw light on the therapeutic potential of reversibly inactivating NADH dehydrogenase via modifying the active/deactive transitions of complex I in ischemic injury.

Ginsenosides, the main bioactive components in *Panax ginseng* C. A. Mey, are widely used to treat cardiovascular diseases. Ginsenoside Rb1, the most abundant ginsenoside in ginseng, is reported to protect the heart against I/R injury 19, 20. Previous studies observed that ginsenoside Rb1 reduces cellular energy charge and ameliorates mitochondrial dysfunction to increase cardiac resistance to I/R injury 21, 22. Ginsenosides have been proposed to share pronounced similarities with metformin from the aspect of metabolic regulation 23. Given the mild cellular energy suppression by metformin, we speculated that regulation of mitochondrial function might be a way for ginsenoside Rb1 to protect the heart against ischemic injury. In this study, through TMT-based proteomic analysis, siRNA knockdown, and rescue assays using yeast NADH dehydrogenase, we identified subunits of NADH dehydrogenase in mitochondrial complex I as the potential proteins regulated by ginsenoside Rb1. Unlike rotenone, ginsenoside Rb1 transiently and reversibly inhibited NADH dehydrogenase activity, retaining complex I in the deactive state to limit ROS production upon reperfusion. These findings provided new insights into the role of ginsenoside Rb1 in cardioprotection and suggested reversible regulation of complex I to be of therapeutic relevance in the prevention of I/R injury.

## Methods

### Materials

Ginsenoside Rb1 (purity >98%) was obtained from a commercial source (Nanjing Spring & Autumn Biological Engineering Co., Ltd., Nanjing, China). Rotenone (R8875), dimethyl succinate (V900547), oligomycin (495455), and MitoB (SML1300) were purchased from Sigma (St. Louis, MO, USA). d_15_-MitoB (17470) and d_15_-MitoP (19296) were purchased from Cayman Chemical (Ann Arbor, MI, USA). Antibodies including anti-Ndufv1 (11238-1-AP), anti-Ndufv2 (15301-1-AP), anti-Ndufs1 (12444-1-AP), anti-Ndufs4 (15849-1-AP), anti-Ndufs6 (14417-1-AP), anti-Ndufa12 (15793-1-AP), anti-Bax (60267-1-Ig), and anti-Bcl2 (26593-1-AP) were purchased from ProteinTech (Chicago, IL, USA). Anti-prohibitin (ab75766) and anti-8 hydroxyguanosine (ab62623) antibodies were purchased from Abcam (Cambridge, MA, USA). Anti-beta-actin (bs-0061R) antibody was purchased from Bioss (Beijing, China). Antibodies such as anti-Bak (BS1029), anti-histone H3 (BS1174), anti-GAPDH (AP0063), goat anti-rabbit IgG (H+L) HRP (BS13278), and goat anti-mouse IgG (H+L) HRP (BS12478) were purchased from Bioworld Technology (St. Paul, MN, USA).

### Animals

C57BL/6J male mice (6-8 or 8-12 weeks) were provided by the Laboratory Animal Center of Nanjing Qinglongshan (Nanjing, China). The mice were fed standard mouse diet ad libitum. Animals were randomized for treatment. Data collection and evaluation of the various groups were blinded. The animal care and experimental procedures were approved by the Animal Ethics Committee of China Pharmaceutical University.

### Cell culture

Primary cardiomyocytes were isolated from adult mice (8-12 weeks) following a previous method 24. Briefly, the chest cavity of the anaesthetized mouse was opened to expose the still-beating heart. The heart was perfused with EDTA buffer through left ventricular injection. Afterward, the heart was transferred into a dish and serially perfused with EDTA buffer, perfusion buffer, and collagenase buffer. The tissue was then separated and gently pulled into 1 mm^3^ pieces using forceps. The cell suspension was passed through a filter, and the cells were collected by gravity with serial reintroduction of calcium buffers to gradually restore calcium concentration. The cells were resuspended in plating media for 1 h at 37 °C in 5% atmosphere. After washing off unattached cells, the remaining cells were incubated in fresh culture media. For hypoxia-reoxygenation (H/R) treatment, cells were subjected to hypoxia (1% O_2_) for 1 h with or without 10 μM ginsenoside Rb1 (this concentration was chosen based on our previous study 21), followed by reoxygenation. For succinate treatment, cardiomyocytes were incubated in assay buffer (132 mM NaCl, 10 mM HEPES, 4.2 mM KCl, 1 mM MgCl_2_, 1 mM CaCl_2_, 25 μM 2-deoxyglucose, 10 mg/L sodium pyruvate; pH 7.4) and subjected to indicated agents in the presence or absence of dimethyl succinate (5 mM) or oligomycin (4 μM) for 2 h.

### *In vivo* myocardial I/R model

A murine model of I/R was generated according to a previously reported method 25. In brief, male C57BL/6J mice were given ginsenoside Rb1 (50 mg/kg, i.p.) and then subjected to 30 min occlusion of the left anterior descending coronary artery followed by either 15 min, 24 h, 14 days, or 28 days of reperfusion. The dosage of ginsenoside Rb1 was selected based on our previous study 21, 26. Hearts were isolated after 15 min of reperfusion for determination of ROS production and proteomics analysis. After 24 h of reperfusion, hearts were isolated for immunohistochemistry examination. Cardiac function assay and transcriptomic analysis were conducted after 14 days of reperfusion. After 28 days of reperfusion, cardiac function was measured prior to heart removal for Masson's trichrome staining analysis.

In addition, to observe the effects of ginsenoside Rb1 administration at different time points, mice were subjected to ligation of the left anterior descending coronary artery for 30 min followed by reperfusion. Mice received ginsenoside Rb1 (50 mg/kg) either before ischemia (i.p.), at the onset of reperfusion (i.v.), or 15 min post-reperfusion (i.p.). After 24 h of reperfusion, hearts were isolated for 2,3,5-triphenyltetrazolium chloride (TTC) staining.

### Measurement of mitochondrial ROS *in vitro* and* in vivo*

For the *in vitro* mitochondrial ROS production assay after H/R treatment, cardiomyocytes were loaded with 5 μM MitoSOX Red mitochondrial superoxide indicator (Molecular Probes, Eugene, OR) for 0.5 h at 37 °C. After washing, ROS production was measured by a fluorescence microplate reader.

Changes in heart mitochondrial ROS in mice subjected to I/R injury were estimated from conversion of the radiometric probe MitoB to MitoP 27. Briefly, 25 nmol MitoB in 100 μL saline was administered via tail vein injection 4 h before initiating ischemia. After 30 min of ischemia (with or without ginsenoside Rb1 pretreatment) followed by reperfusion for 15 min, 14 days, or 28 days, hearts were removed and flash frozen in liquid nitrogen. Heart tissue samples were homogenized, spiked with deuterated internal standards (d_15_-MitoB and d_15_-MitoP), and MitoB and its product MitoP were extracted. The amounts of MitoB and MitoP were determined by liquid chromatography and tandem mass spectrometry (LC-MS/MS, LCMS8050, Shimadzu, Japan).

### TMT-based quantitative proteomics

Harvested hearts were stored immediately in liquid nitrogen. Mitochondria were extracted and purified from the left ventricle using Mitochondria Isolation Kit for Tissue (Beyotime). The purified mitochondrial samples were homogenized in lysis buffer to extract mitochondrial proteins. The global proteome was analyzed at PTM Biolabs Inc. (Hangzhou, China). In brief, protein concentration and purity of the samples were quantified. The samples were then digested with trypsin, labeled with a TMT kit (Thermo Fisher Scientific Incorporation, MA, USA), and fractionated by high-pH reverse-phase high-performance liquid chromatography using an Agilent 300 Extend C18 column. The peptides were divided into 18 fractions, dried by vacuum centrifugation, and analyzed by LC-MS/MS. The resulting MS/MS data were processed using Maxquant search engine (v.1.5.2.8). Tandem mass spectra were searched against Swiss-Prot Mouse database concatenated with reverse decoy database. Trypsin/P was specified as cleavage enzyme allowing up to 2 missing cleavages. The minimum length of the peptide was set to 7 amino acid residues, and the maximum number of modifications for peptides was 5. The mass tolerance for precursor ions was set as 20 ppm in First search and 5 ppm in Main search, and the mass tolerance for fragment ions was set as 0.02 Da. Carbamidomethyl on cysteine was specified as fixed modification, and oxidation on methionine and acetylation of the protein N-terminal were specified as variable modifications. TMT-9plex was set as quantitative method. False discovery rates for protein and peptides identifications were adjusted to <1% and minimum score for peptides was set >40. The subcellular localization of identified proteins was annotated using WoLFPSORT database (https://www.genscript.com/wolf-psort.html). A *p*-value <0.05 between comparative groups was utilized to identify differentially expressed proteins. Cluster membership was visualized by a heat map using the “heatmap.2” function from the “gplots” R-package. To perform functional analysis, the identified proteins were analyzed using Gene Ontology (GO) Terms with UniProt-GOA database (http://www.ebi.ac.uk/GOA/). Pathway mapping of identified proteins was performed using the KEGG database (http://www. genome.jp/kegg/). Briefly, the identified proteins were mapped to the KEGG database. The enriched pathways were extracted and analyzed by a two-tailed Fisher's exact test to determine the enrichment of the differentially expressed proteins against all identified proteins. Pathways with a corrected *p*-value <0.05 were considered significant. Protein-protein interactions were analyzed using the STRING database. The mass spectrometry proteomics data have been deposited in the ProteomeXchange Consortium via the PRIDE 28 partner repository with the dataset identifier PXD011414.

### Transcriptome profiling via mRNA-Seq

Heart RNA was isolated using RNeasy Plus Kit with the genomic DNA removal step. The concentration and quality of extracted RNA were evaluated. cDNA library construction and sequencing were performed by Annoroad Gene Technology (Beijing, China). The raw sequencing reads were cleaned by removing adaptors and low-quality reads (reads with a Q-value <20). After filtering, Bowtie software 29 was used to map the clean reads to the mouse (vM16) reference genome (2.6 G). The software was downloaded from the University of California, Santa Cruz (UCSC) website (http://hgdownload.soe.ucsc.edu/hubs/mouseStrains/hubIndex.html). Gene expression was quantified by calculating fragments per kilobase of exon per million fragments mapped (FPKM) values using the STAR software package. Significantly changed genes were selected to determine significant differences in gene expression between control and experimental groups. The raw data from the transcriptomic experiment have been deposited in the SRA database of NCBI (SRA accession: PRJNA552785).

### Oxygen consumption rate (OCR) assay

OCR measurements were performed using XF96 Extracellular Flux Analyzer (Agilent technologies, USA). H9c2 cells were seeded (10000 cells/well) in cell culture plates. On the day of the assay, the cells were subjected to mannitol and sucrose buffer (70 mM sucrose, 220 mM mannitol, 10 mM KH_2_PO_4_, 5 mM MgCl_2_, 2 mM HEPES, 1 mM EGTA, 0.2% (w/v) fatty acid-free BSA; pH 7.2). OCR measurements were conducted, and drugs were injected sequentially as indicated. After permeabilization by digitonin, substrates and inhibitors of mitochondrial OXPHOS were added at the following concentrations: ADP (1 mM), pyruvate (5 mM)/malate (5 mM), glutamate (5 mM), succinate (5 mM), rotenone (1 μM), TMPD (0.4 mM)/ascorbate (0.4 mM), FCCP (2.5 μM), antimycin A (1 μM) and azide (5 mM). Among these treatments, the substrate mixture of complex I-dependent respiration consisted of pyruvate/malate or glutamate/malate and was followed by addition of ADP. The combination for succinate/rotenone activated by ADP was used to assay complex II-associated respiration. TMPD in combination with ascorbate was used to induce complex IV-dependent respiration followed by addition of complex IV inhibitor azide. FCCP was used to measure uncoupled respiration. Antimycin A, an inhibitor of complex III, was added to inhibit oxygen consumption induced by mitochondrial electron transport chain (ETC).

### Measurement of mitochondrial complex activities

H9c2 cells were harvested to extract mitochondrial proteins. Equal amounts of protein from each group were used to measure the activity of mitochondrial complexes using commercial kits (GENMED Scientifics, Inc., Shanghai, China). Complex I activity was determined by the rotenone-sensitive oxidation of NADH. Oxidation of ferrocytochrome c by complex IV and succinate:Q_1_-reductase activity of complex II were measured. In addition, NADH dehydrogenase enzyme activity that is rotenone-insensitive was determined using Mitochondrial OXPHOS Complex I (NADH dehydrogenase) Enzyme Activity Kit (Abcam, Cambridge, MA, USA). Correlations between NADH dehydrogenase activity and the protein levels of subunits in NADH dehydrogenase, which were the differential proteins identified by proteomics in Table 1, were analyzed using the R language (R 3.6.0) (https://cran.r-project.org/).

### Quantitative real-time PCR

After hypoxia for 1 h followed by reperfusion for 15 min with or without ginsenoside Rb1 pretreatment, total RNA was isolated from cardiomyocytes using TRIeasy^TM^ Reagent (Yeasen, Shanghai, China). After evaluating RNA concentration and quality, the extracted RNA was reversely transcribed into cDNA using Hieff™ First Strand cDNA Synthesis Super Mix (Yeasen). Relative gene expressions were quantified by Hieff™ qPCR SYBR Green Master Mix Kit (Yeasen) with CFX96^TM^ Real-Time PCR System (BIO-RAD, USA). mRNA levels were calculated using the 2^-ΔΔCt^ method and were presented as a ratio to β-actin. Primer sequences were listed in Table S1.

### Small interfering RNA transfection and yeast NADH dehydrogenase (Ndi1)-expressing construct

H9c2 cells were transfected with Ndufv1, Ndufv2, Ndufs1, Ndufs4, Ndufs6, or Ndufa12 siRNA (GenePharma, Shanghai, China). The siRNA sequences were listed in Table S2. At 48 h post-transfection, the cells were subjected to hypoxia and reoxygenation, and ROS production and cell viability were determined.

Ndi1 plasmid was obtained from Genomeditech Biotechnology (Shanghai, China). Yeast genomic DNA was extracted from wild type *Saccharomyces cerevisiae* using a yeast DNA extraction kit. The Ndi1 gene was PCR-amplified from yeast genomic DNA using primers (F: 5′ TGTAAAACGACGGCCAGT 3′ and R: 5′ CCGCTCGAGTAATCCTTTAAAAAAGTCTCTTTTGAAAAATGCTAATTTAATCC 3′). After digestion with EcoRI and XhoI, PCR products were ligated into the PGMLV-CMV-MCS-3*flag-EF1-ZsGreen1 vector and confirmed by DNA sequencing. The Ndi1 plasmids were then purified using Plasmid DNA Purification Kit (QIAGEN, Germany). Transfection efficiency was determined by Western blot with anti-Flag antibody (20311007, Bioworld Technology Co., Ltd., MN, USA). Then, OCR was measured in H9c2 cells treated with ginsenoside Rb1. The transfected cells were also subjected to hypoxia and reoxygenation or succinate treatment and ROS production was evaluated.

### Fluorescent labeling of ND3 subunit

Mitochondria were isolated from the hearts of normal C57BL/6J mice using Mitochondria Isolation Kit for Tissue (Beyotime). The mitochondrial proteins were divided into 2 parts. One part was incubated at 37 °C for 45 min on a dry heating block with or without ginsenoside Rb1 (10 μM) to induce deactive complex I. Subsequently, reactivation of the sample was achieved by adding NADH (10 μM) at RT for 2 min. The other part was kept on ice in the presence or absence of ginsenoside Rb1. After treatment, the samples were incubated with BODIPY-TMR C5-maleimide (Invitrogen, USA) at 15 °C for 20 min in the dark, followed by further incubation with cysteine (1 mM) for 5 min. The samples were then precipitated with acetone to obtain the protein. For heart samples from I/R-treated mice, mitochondria were isolated and immediately subjected to BODIPY-TMR C5-maleimide incubation and subsequent treatment. The proteins were separated by SDS-PAGE to detect the fluorescence signals. Total protein was assessed by Sypro Ruby staining (Invitrogen). Images were obtained using a digital fluorescent image analyzer (Tanon, Shanghai, China). The protein band around 10 kDa was cut and analyzed by LC-MS/MS.

### Molecular docking

The protein structure of human respiratory complex I (Protein Data Bank ID 5XTD) was downloaded from the Protein Data Bank (https://www.rcsb.org/). The docking conformation of ginsenoside Rb1 and subunits involved in active/deactive transition (ND3, ND1, and Ndufa9) was simulated using AutoDock (version 4.2.6). The grid box was set to the dimensions of 80 × 80 × 80 Å with grid spacing of 0.375 Å. 150 individual genetic algorithm runs were executed to obtain 150 docking conformations to select the optimal conformation.

### Surface plasmon resonance (SPR) analysis

The binding affinity of ginsenoside Rb1 to ND3 protein was measured using a Biacore T200 (GE Healthcare). Human recombinant ND3 protein (APREST88027, Sigma) was captured on a CM5 chip by a standard amine coupling procedure. Binding sensorgrams were recorded by injecting various concentrations of ginsenoside Rb1 (0.1, 0.2, 0.39, 0.78, and 1.56 μM) over the immobilized ND3 surface. The data were fitted and analyzed to obtain the equilibrium dissociation constant (K_D_) value.

### Mitochondrial pH measurement

Mitochondrial matrix pH was detected by a mitochondria-targeted pH-sensitive probe mito-SypHer (GenTarget Inc., CA, USA). H9c2 cells were stably transfected with mito-SypHer (CMV-Puro) lentivirus. The positively transduced cells were sorted and subjected to heat-induced expression, and then subcellular localization of mito-SypHer was validated by confocal microscopy. The cells were then subjected to hypoxia or H/R insult with or without ginsenoside Rb1 (10 μM) treatment. The ratio of fluorescence signals from excitation at 480 nm and 420 nm was recorded.

### Western blot assay

After treatment, cardiomyocytes were lysed in ice-cold RIPA buffer to obtain protein. Equivalent amounts of protein were separated by SDS-PAGE and transferred onto PVDF membranes and then blocked at RT for 3 h. The membranes were immunoblotted with primary antibodies at 4 °C overnight, followed by incubation with HRP-conjugated secondary antibody. The blots were visualized using ECL and quantified by ImageProPlus 6.0 software.

### Mitochondrial function and cell viability analysis

Primary cardiomyocytes were subjected to hypoxia for 1 h followed by 1 h reperfusion with or without ginsenoside Rb1 pretreatment. The time point was selected with reference to previous reports 10, 30. After H/R treatment, the conditioned medium was collected for a lactate assay (Jiancheng Bioengineering Institute, Nanjing, China) and the cells were harvested for ATP contents detection by ATP Assay Kit (Beyotime Institute of Biotechnology) according to the manufacturer's instructions. For the mitochondrial membrane potential (Δψm) assay, treated cardiomyocytes were loaded with a fluorescent indicator tetramethylrhodamine ethyl ester (TMRE) for 30 min at 37 °C. TMRE staining was examined by confocal microscopy (Zeiss LSM 700). MPTP opening was assayed with Mitochondrial Transition Pore Assay Kit (Life Technology, USA) according to the manufacturer's protocol. Cell viability was measured using Cell Counting Kit-8 (CCK-8) (Yeasen).

### Statistical analysis

The data were expressed as mean ± SD. All experiments were performed at least three times. Significant differences were analyzed by one-way ANOVA followed by Bonferroni correction using GraphPad Prism 7. *p*-value <0.05 was considered statistically significant.

## Results

### Ginsenoside Rb1 reduces succinate-driven ROS production upon reperfusion

**Figure 1:**
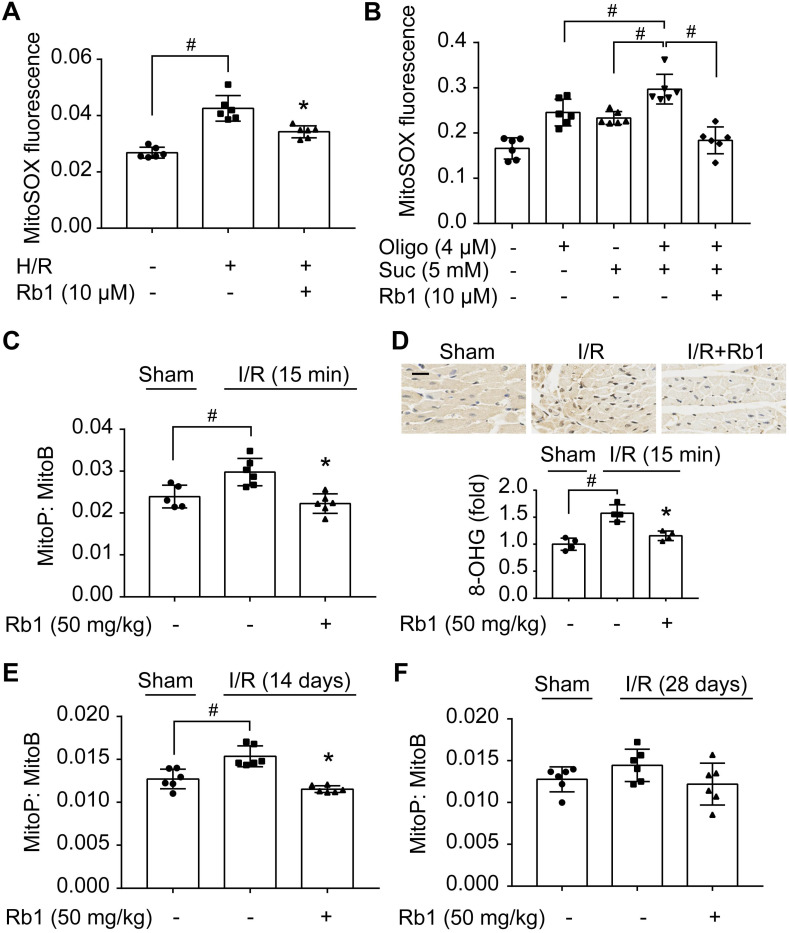
** Ginsenoside Rb1 reduces succinate-driven ROS production upon reperfusion. A**, ROS production assessed by MitoSOX Red mitochondrial superoxide indicator in adult primary cardiomyocytes subjected to hypoxia/reoxygenation treatment. **B**, ROS production in primary cardiomyocytes subjected to dimethyl succinate (Suc) and oligomycin (Oligo) treatment for 2 h. **C**, Inhibitory effect of ginsenoside Rb1 on ROS generation as assessed by MitoB oxidation 15 min post-reperfusion* in vivo* (n = 5, 6). **D**, Immunohistochemistry examinations of 8-hydroxyguanosine (8-OHG) in hearts from mice subjected to ischemia (30 min) followed by 15 min reperfusion (n = 4). Scale bar, 20 µm. **E-F**, Effect of ginsenoside Rb1 pretreatment on heart ROS levels at days 14 (E) or 28 (F) post-reperfusion (n = 6). Data were expressed as mean ± SD. ^*^p < 0.05: *vs.* H/R or I/R only treatment; ^#^*p* < 0.05: *vs.* indicated treatment. H/R, hypoxia/reoxygenation; I/R, ischemia/reperfusion; Rb1, ginsenoside Rb1.

Mitochondrial ROS rapidly increased upon H/R insult in primary adult mouse cardiomyocytes but was halted by pretreatment with ginsenoside Rb1 (Figure 1A). Because accumulated succinate is a driving force for mitochondrial ROS production upon reperfusion 10, we treated cardiomyocytes with cell-permeable dimethyl succinate with ATP synthase inhibition by oligomycin 31 to mimic RET-mediated ROS production. Succinate fueled extensive ROS production due to the high Δψm induced by ATP synthase inhibition, which was reduced by pretreatment with ginsenoside Rb1 (Figure 1B). To confirm its role *in vivo*, we used MitoB, a mitochondria-targeted ROS probe 27, to quantify ROS production in the hearts of mice subjected to I/R insult. The results showed that ginsenoside Rb1 administration effectively blocked mitochondrial ROS production during the early reperfusion phase (Figure 1C). Consequently, ginsenoside Rb1 attenuated oxidative DNA damage (Figure 1D). In addition, we observed that ginsenoside Rb1 administration before reperfusion reduced ROS levels significantly at the 14^th^ day after I/R surgery and this effect remained even at day 28 (Figure 1E-F).

### Attenuation of ROS burst by ginsenoside Rb1 protects cardiomyocytes from acute I/R injury

**Figure 2:**
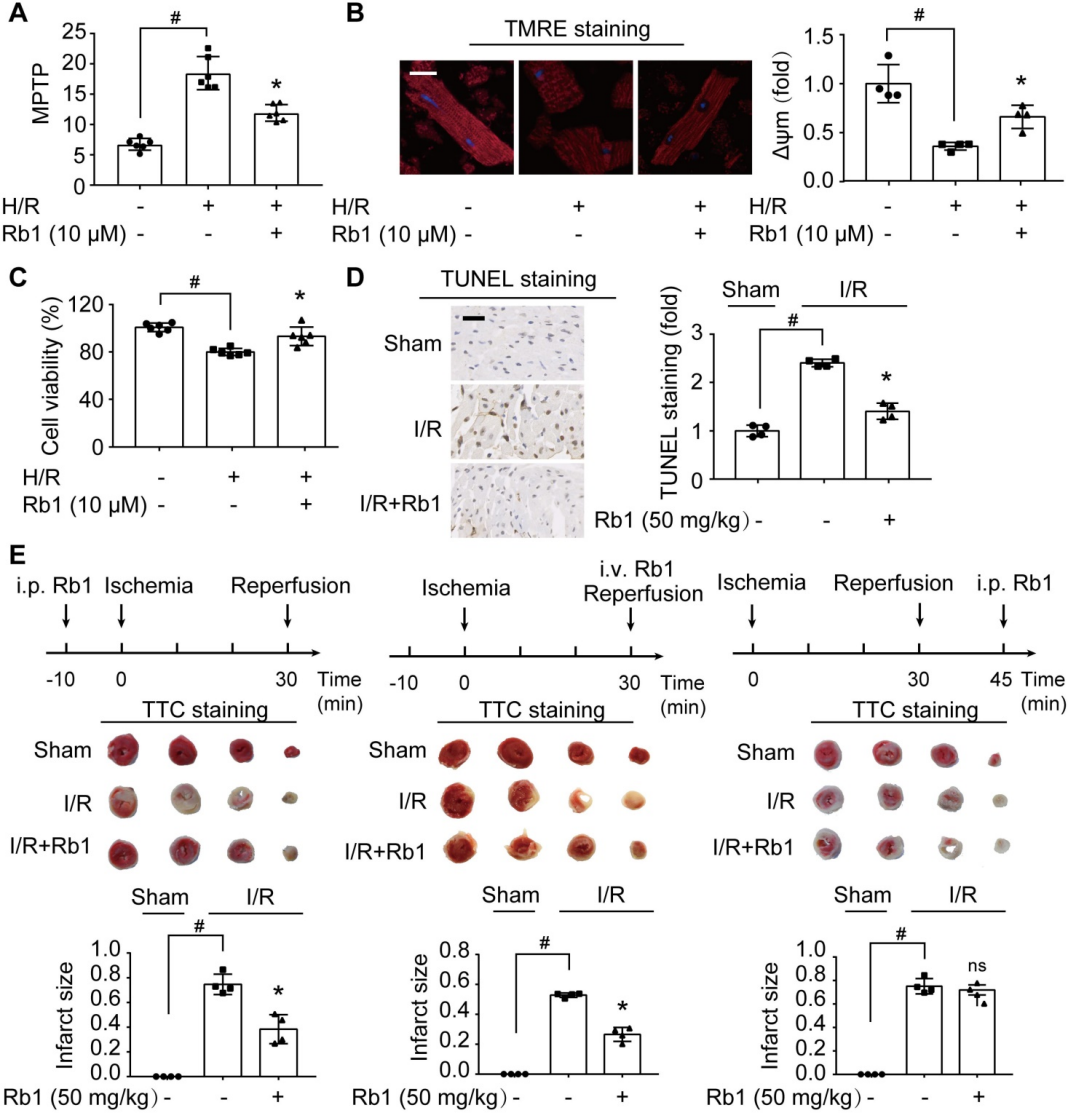
** Attenuation of ROS burst by ginsenoside Rb1 protects cardiomyocytes from acute I/R injury. A-C**, Adult primary cardiomyocytes were treated with ginsenoside Rb1 (10 µM) and subjected to hypoxia (1% O_2_) for 1 h followed by 1 h reoxygenation (H/R). Mitochondrial permeability transition pore (A), representative images and quantification of mitochondrial potential (Δψm) from four independent experiments (B, bar: 20 µm), and cell viability (C) were assayed.** D**, Representative images and quantification of TUNEL staining of hearts from mice subjected to ischemia (30 min) followed by 24 h reperfusion (n = 4). Scale bar, 50 µm. **E**, Myocardial infarct size in mice subjected to cardiac ischemia and reperfusion injury with ginsenoside Rb1 administered prior to ischemia, at the onset of reperfusion, or 15 min post-reperfusion (n = 4). Representative photographs of TTC staining 24 h post-reperfusion. Data were expressed as mean ± SD. ^*^p < 0.05: *vs.* H/R only treatment or I/R only treatment; ^#^*p* < 0.05: *vs.* indicated group; ns. no significance: *vs.* I/R only treatment. H/R, hypoxia/reoxygenation; I/R, ischemia/reperfusion; Rb1, ginsenoside Rb1; TMRE, tetramethylrhodamine ethyl ester; TTC, 2,3,5-triphenyltetrazolium chloride.

H/R insult impaired mitochondrial function, as indicated by opening of MPTP and collapse of Δψm in primary cardiomyocytes (Figure 2A-B). Consequently, the mitochondrial apoptotic pathway was activated, evidenced by upregulation of Bax and Bak protein expressions with downregulation of Bcl2 (Figure S1A-C). These alterations were prevented by ginsenoside Rb1 pretreatment. Concordantly, ginsenoside Rb1 attenuated cell death (Figure 2C). Meanwhile, ginsenoside Rb1 reduced lactate accumulation with the restoration of ATP contents in cardiomyocytes (Figure S1D-E). Similarly, ginsenoside Rb1 administration prior to I/R insult effectively reduced myocardial apoptosis (Figure 2D). Administration of ginsenoside Rb1 both before ischemia and at the onset of reperfusion reduced infarct size significantly (Figure 2E). However, this protective effect was not observed when it was administered 15 min post-reperfusion (Figure 2E), indicating that timely intervention is critical in cardioprotection.

### Ginsenoside Rb1 administration before reperfusion contributes to improving cardiac function during the recovery process

**Figure 3:**
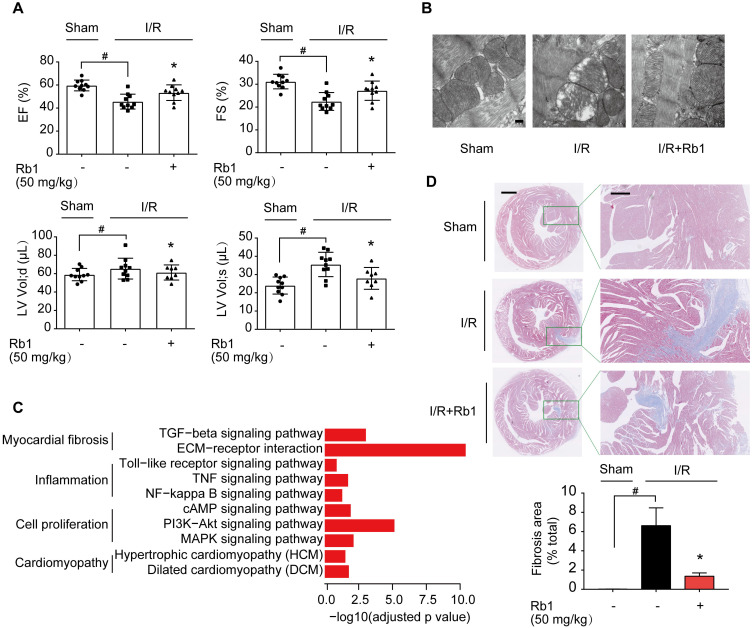
** Ginsenoside Rb1 administration before reperfusion contributed to improving cardiac function during the recovery process. A**, Heart function measured 14 days post-I/R insult (n = 7-10). **B**, Mitochondrial structure of the heart assayed 14 days post-I/R insult (n = 3). Scale bar, 200 nm. **C**, Cardiac gene profiling measured 14 days post-I/R insult (n = 3). **D**, Representative images and quantification of Masson's trichrome staining in hearts from mice 28 days post-I/R insult (n = 3). Scale bars, 1 mm (left) and 300 µm (right). Data were expressed as mean ± SD. ^*^p < 0.05: *vs.* I/R only treatment; ^#^*p* < 0.05: *vs.* indicated group. I/R, ischemia/reperfusion; EF, ejection fraction; FS, fractional shortening; LV vol; d, left ventricular end-diastolic volume; LV vol; s, left ventricular end-systolic volume; Rb1, ginsenoside Rb1.

ROS production during reperfusion is responsible for impaired cardiac function with poor prognosis 6. We observed that ginsenoside Rb1 administration prior to ischemia preserved cardiac function and prevented mitochondrial swelling in mice 2 weeks post-I/R insult (Figure 3A-B). Corresponding results from cardiac gene profiling revealed that several biological pathways related to myocardial fibrosis were affected. These gene sets were related to the TGF-β signaling pathway and ECM-receptor interaction, and their upregulation was reversed by ginsenoside Rb1 administration prior to reperfusion (Figure 3C, Figure S2A). As the heart injury progressed, ginsenoside Rb1 administration before ischemia preserved cardiac function and attenuated cardiac fibrosis in mice examined 28 days after I/R insult (Figure S2B, Figure 3D). These results indicated that administration of ginsenoside Rb1 before reperfusion not only decreased acute cardiomyocytes damage but also contributed to improving cardiac function during the recovery process.

### Ginsenoside Rb1 regulates mitochondrial complex I as revealed by proteomics

**Figure 4:**
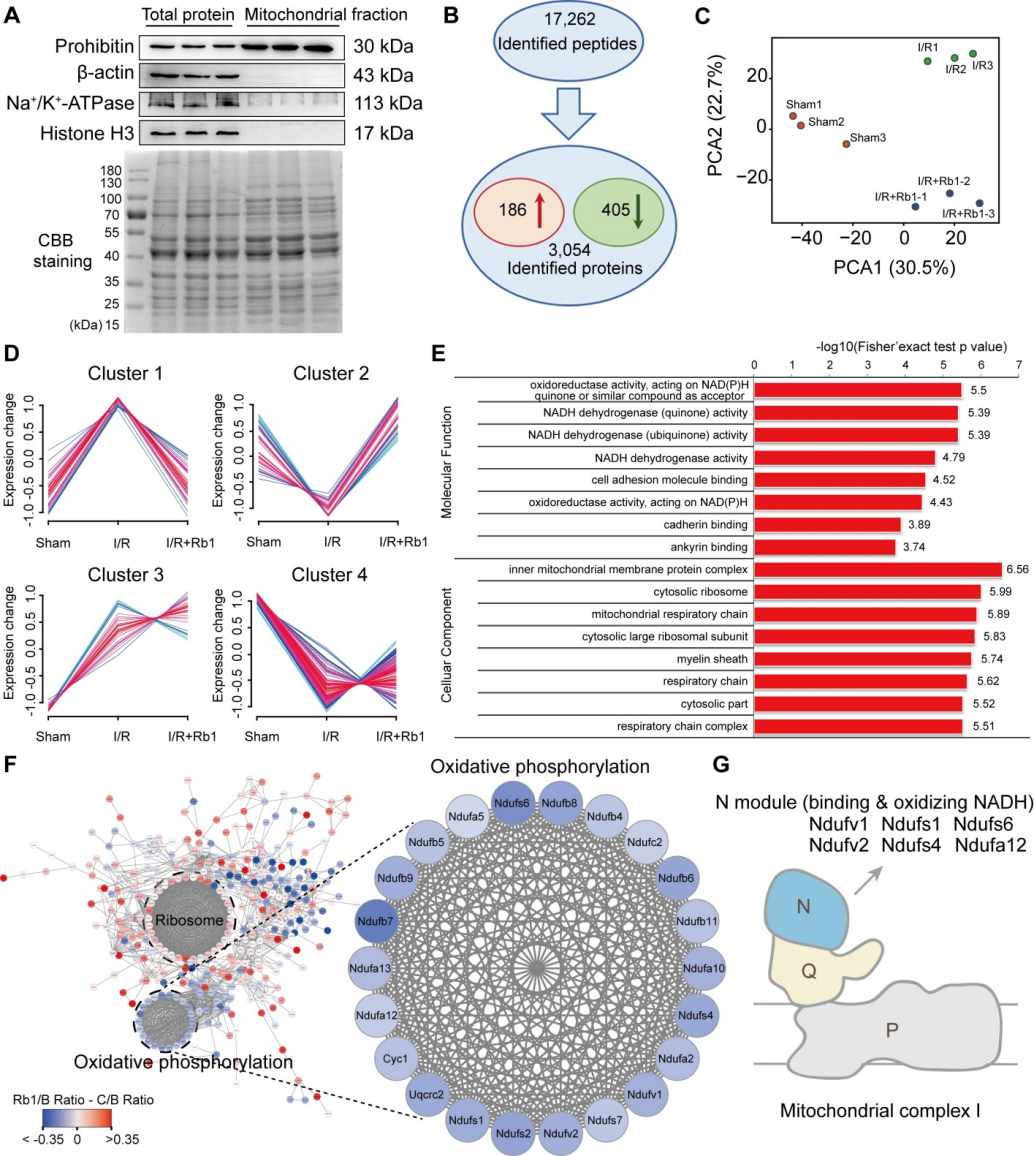
** Ginsenoside Rb1 regulates mitochondrial complex I as revealed by proteomics.** C57BL/6J mice were given ginsenoside Rb1 (50 mg/kg, i.p.) and subjected to cardiac ischemia (30 min) followed by 15 min of reperfusion. Mitochondrial proteins of the heart tissue were extracted for proteomic analysis. **A**, Western blot assay to check the purity of enriched mitochondrial fractions obtained from heart tissues (n = 3). **B**, Significantly changed proteins identified from comparison of ischemia/reperfusion (I/R) and sham groups. **C**, Principal component analysis (PCA) of sham group (Sham), ischemia/reperfusion group (I/R), and I/R plus ginsenoside Rb1 treated group (I/R+Rb1) based on identified proteins (n = 3). **D**, Cluster analysis of significantly changed proteins in Sham, I/R, and I/R+Rb1 groups based on their change tendencies. Each fold line indicated a change tendency of one protein. Proteins positioned in Clusters 1 and 2 were recognized as rescued or regulated by ginsenoside Rb1. **E**, Functional categorization of proteins rescued or regulated by ginsenoside Rb1 using the Gene Ontology (GO) database. **F**, Protein-protein interaction analysis of proteins rescued or regulated by ginsenoside Rb1 using the STRING database. Red and blue indicated degree of protein rescued or regulated by ginsenoside Rb1. **G**, Schematic diagram of the distribution of proteins positioned in mitochondrial complex I rescued or regulated by ginsenoside Rb1 treatment.

Because mitochondrial ROS is responsible for reperfusion injury, we purified mitochondria from the hearts of mice subjected to I/R insult for TMT-based quantitative proteomic analysis. The relative intensity of prohibitin was significantly increased in the mitochondrial protein fraction with an absence of other proteins, an indication of its high purity and significant enrichment (Figure 4A). With reference to these qualified data (Figure S3A-B), 17262 peptides (with a confidence level ≥ 95%) were mapped to 3054 protein groups (Figure 4B). Among them, mitochondrial proteins accounted for nearly 50% (Figure S3C, Table S3), indicating their high enrichment and predominance in the fractions. With a criterion of *p*-value <0.05, 591 significantly changed proteins were found. Among them, 186 were elevated while 405 were decreased in the I/R group compared with the sham group (Figure 4B). PCA analysis showed that the I/R group was clearly separated from the sham group, and the protein pattern was partly reversed by ginsenoside Rb1 treatment (Figure 4C).

Next, these differential proteins were divided into 4 clusters based on their change tendencies (Figure 4D). Proteins in Cluster 1 were highly increased in the I/R group but clearly decreased in the ginsenoside Rb1-treated group. Proteins in Cluster 2 were decreased in the I/R group but increased in the ginsenoside Rb1-treated group. Proteins in Clusters 3 and 4 were not significantly influenced by ginsenoside Rb1 treatment. Based on these results, proteins positioned in Clusters 1 and 2 were recognized as rescued or regulated by ginsenoside Rb1. GO analysis of Clusters 1 and 2 indicated that the molecular functions of these proteins were closely related to oxidoreductase and NADH dehydrogenase activities (Figure 4E). Interestingly, protein-protein interaction analysis showed that these proteins interacted with each other and were associated with oxidative phosphorylation and ribosome functions (Figure 4F). Importantly, KEGG enrichment analysis showed that the proteins in the oxidative phosphorylation part were mainly related to mitochondrial ETC function (Figure S3D). Specifically, mitochondrial complex I proteins were upregulated in myocardial injury, but rescued to normal levels by ginsenoside Rb1 (Figure 4F). Among them, proteins positioned in subunits of NADH dehydrogenase (N module) such as Ndufv1, Ndufv2, Ndufs1, Ndufs4, Ndufs6, and Ndufa12 were significantly rescued by ginsenoside Rb1 treatment (Figure 4G, Table 1). Taken together, these results suggest that ginsenoside Rb1 protected the heart from I/R injury possibly by regulating proteins positioned in mitochondrial complex I.

### Ginsenoside Rb1 selectively inhibits the activity of mitochondrial complex I but not complexes II and IV

**Figure 5:**
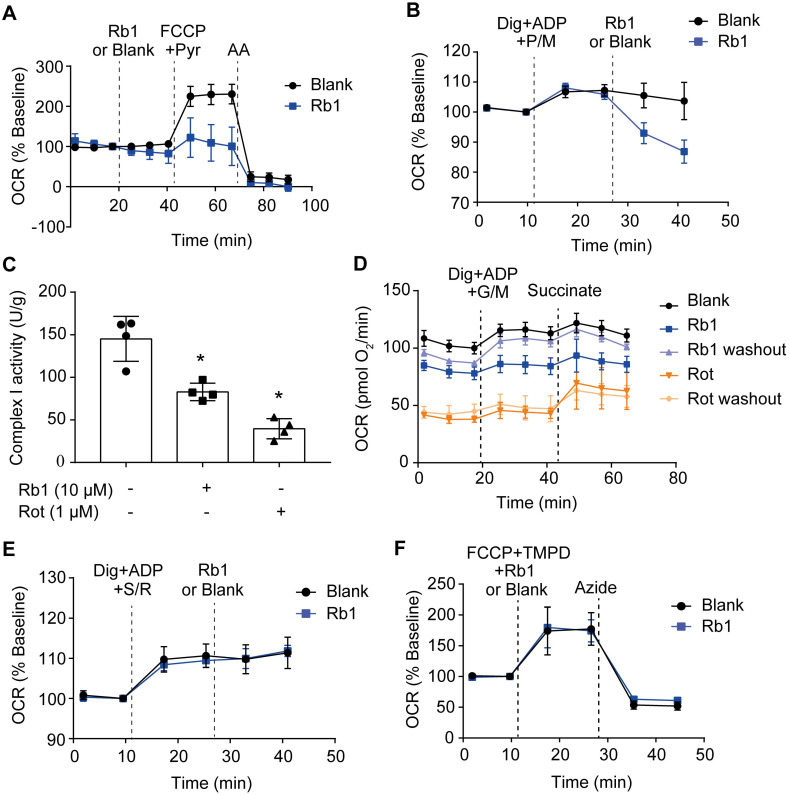
** Ginsenoside Rb1 selectively inhibits the activity of mitochondrial complex I but not complexes II and IV. A**, Oxygen consumption rate (OCR, % baseline) of H9c2 cells in the presence of ginsenoside Rb1 (10 µM), FCCP (2.5 µM), pyruvate (Pyr, 10 mM), and antimycin A (AA, 1 µM). **B**, OCR traces of H9c2 cells permeabilized by digitonin (3 µg/mL) and then stimulated by ADP (1 mM), pyruvate (5 mM), malate (5 mM) and Rb1 (10 µM). **C**, Mitochondrial complex I activity in H9c2 cells treated with ginsenoside Rb1 (10 µM) or rotenone (1 µM). **D**, OCR traces of permeabilized H9c2 cells exposed to ADP (1 mM), glutamate (5 mM) plus malate (5 mM), and succinate (5 mM). Here, H9c2 cells were pretreated with ginsenoside Rb1 (10 µM) or rotenone (1 µM) for 1 h prior to OCR measurements. Then ginsenoside Rb1 or rotenone was then either left on for further assay, or washed out with replacement of drug-free assay buffer. **E**, OCR (% baseline) of permeabilized H9c2 cells in the presence of succinate (5 mM), rotenone (0.5 µM), and ginsenoside Rb1 (10 µM). **F**, OCR (% baseline) of H9c2 cells injected with FCCP (2.5 µM), TMPO (0.4 mM) plus ascorbate (0.4 mM), ginsenoside Rb1 (10 µM), and azide (5 mM). Data were expressed as mean ± SD. ^*^p < 0.05:* vs.* untreated control. AA, antimycin A; Dig, digitonin; FCCP, carbonyl cyanide p-trifluoromethoxyphenylhydrazone; G, glutamate; M, malate; Pyr or P, pyruvate; Rb1, ginsenoside Rb1; Rot or R, rotenone; S, succinate; TMPD, tetramethyl-p-phenylene diamine.

Next, we examined whether ginsenoside Rb1 regulates mitochondrial electron transfer chain function. Despite having no significant influence on basal OCR, ginsenoside Rb1 significantly inhibited maximal respiration without affecting antimycin A-insensitive non-mitochondrial O_2_ consumption (Figure 5A). Ginsenoside Rb1 treatment inhibited complex I-dependent O_2_ consumption in cardiomyocytes when pyruvate and malate were used as substrates (Figure 5B). Concordantly, ginsenoside Rb1 inhibited mitochondrial complex I activity under basal conditions in isolated mitochondria (Figure 5C). In contrast to rotenone, which inhibits mitochondrial complex I irreversibly, washing out of ginsenoside Rb1 restored complex I activity (Figure 5D). This result indicated that the inhibitory effect of ginsenoside Rb1 was reversible. Ginsenoside Rb1 did not alter O_2_ consumption in intact cardiomyocytes when succinate was used as a substrate for complex II activation, and it also failed to inhibit mitochondrial complex II activity in isolated mitochondria (Figure 5E, Figure S4A). Similarly, ginsenoside Rb1 had no influence on mitochondrial complex IV-dependent respiration and complex IV activity (Figure 5F, Figure S4B). Taken together, these results demonstrated that ginsenoside Rb1 specifically inhibited mitochondrial complex I activity in a reversible manner.

### Ginsenoside Rb1 reversibly inhibits NADH dehydrogenase of mitochondrial complex I

**Figure 6:**
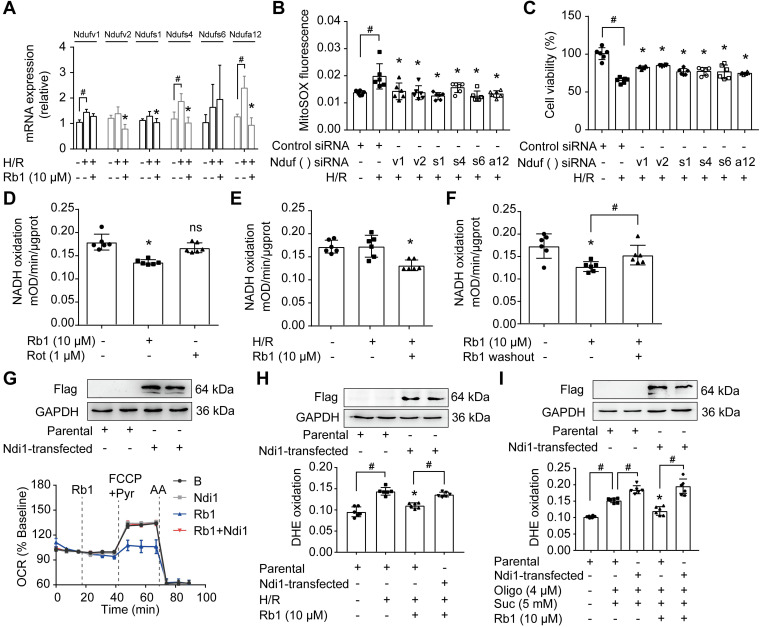
** Ginsenoside Rb1 reversibly inhibits NADH dehydrogenase of mitochondrial complex I. A**, mRNA expressions of Ndufv1, Ndufv2, Ndufs1, Ndufs4, Ndufs6, and Ndufa12 in H/R-treated cardiomyocytes. **B-C**, ROS level (B) and cell viability (C) of H9c2 cells transfected with Ndufv1, Ndufv2, Ndufs1, Ndufs4, Ndufs6, or Ndufa12 siRNA. **D**, NADH dehydrogenase activity in adult primary cardiomyocytes with ginsenoside Rb1 or rotenone treatment. **E**, NADH dehydrogenase activity in adult primary cardiomyocytes upon hypoxia (1 h) followed by 15 min of reperfusion. **F**, Adult primary cardiomyocytes were pretreated with ginsenoside Rb1 (10 µM) for 1 h. Then ginsenoside Rb1 was then either left on for the further assay, or washed out. The culture was continued for another 1 h. NADH dehydrogenase activity was measured. **G**, Ndi1 protein expression in H9c2 cells transfected with Ndi1 plasmid. Oxygen consumption rate (OCR, % baseline) in Ndi1-expressing cells in the presence of ginsenoside Rb1 (10 µM), FCCP (2.5 µM), pyruvate (Pyr, 10 mM) and antimycin A (AA, 1 µM). **H**, Ndi1 protein expression. ROS levels in Ndi1-expressing cells in response to H/R injury. **I**, Ndi1 protein expression. ROS production in Ndi1-expressing cells subjected to dimethyl succinate and oligomycin for 2 h. Data were expressed as mean ± SD. ^*^p < 0.05: *vs.* the untreated control, H/R only treatment or suc plus oligo treatment; ^#^*p* < 0.05: *vs.* indicated treatment; ns. no significance: *vs.* untreated control. H/R, hypoxia/reoxygenation; Oligo, oligomycin; Rb1, ginsenoside Rb1; Rot, rotenone; Suc, succinate.

NADH dehydrogenase of complex I is the candidate site for ROS production 14. Since the proteomic results showed that the levels of differential proteins belonging to subunits of NADH dehydrogenase were altered in response to I/R insult, we examined the gene and protein expressions of these proteins. H/R treatment upregulated mRNA expressions of Ndufv1, Ndufv2, Ndufs1, Ndufs4, and Ndufa12 in cardiomyocytes, but these increased gene expressions were reduced by pretreatment with ginsenoside Rb1 (Figure 6A). We further validated the protein expressions of these subunits in the hearts of mice subjected to I/R insult and confirmed the inhibitory effects of ginsenoside Rb1 in this regard (Figure S5A). Gene knockdown of these subunits of NADH dehydrogenase reduced ROS production and protected cell viability under H/R conditions (Figure 6B-C). Ginsenoside Rb1 reduced NADH dehydrogenase activity of mitochondrial complex I in cardiomyocytes under both basal and H/R conditions (Figure 6D-E). There was a strong correlation between the protein levels of these subunits and NADH dehydrogenase activity (Figure S5B), suggesting that the changes in NADH dehydrogenase activity were regulated at the level of protein-expression. Mitochondrial complex I inhibitor rotenone failed to inhibit NADH dehydrogenase because it binds to ubiquinone rather than NADH dehydrogenase 32. Distinct from rotenone, ginsenoside Rb1 was unable to increase ROS levels and had no influence on cell viability under basal conditions (Figure S6A-B). Washing out of ginsenoside Rb1 restored NADH dehydrogenase activity, indicating that complex I inhibition by ginsenoside Rb1 is reversible (Figure 6F). To confirm the special role of NADH dehydrogenase in the regulation by ginsenoside Rb1, we transfected the gene encoding the internal NADH dehydrogenase enzyme of *Saccharomyces cerevisiae* into H9c2 cells. Ndi1 is a single polypeptide that works as a replacement molecule for complex I in the respiratory chain of mammalian mitochondria 33. Ginsenoside Rb1 failed to reduce OCR in Ndi1-expressing cells (Figure 6G). Consistently, cells expressing Ndi1 were resistant to the effects of ginsenoside Rb1 on ROS production upon H/R stimulation (Figure 6H). Expression of Ndi1 aggravated RET-mediated ROS production and abolished the inhibitory effect of ginsenoside Rb1 (Figure 6I). These results suggested that the cardioprotection by ginsenoside Rb1 may be due to its inhibition of NADH dehydrogenase activity in mitochondrial complex I.

### Ginsenoside Rb1 controls the active/deactive transition of mitochondrial complex I to regulate NADH dehydrogenase activity

**Figure 7:**
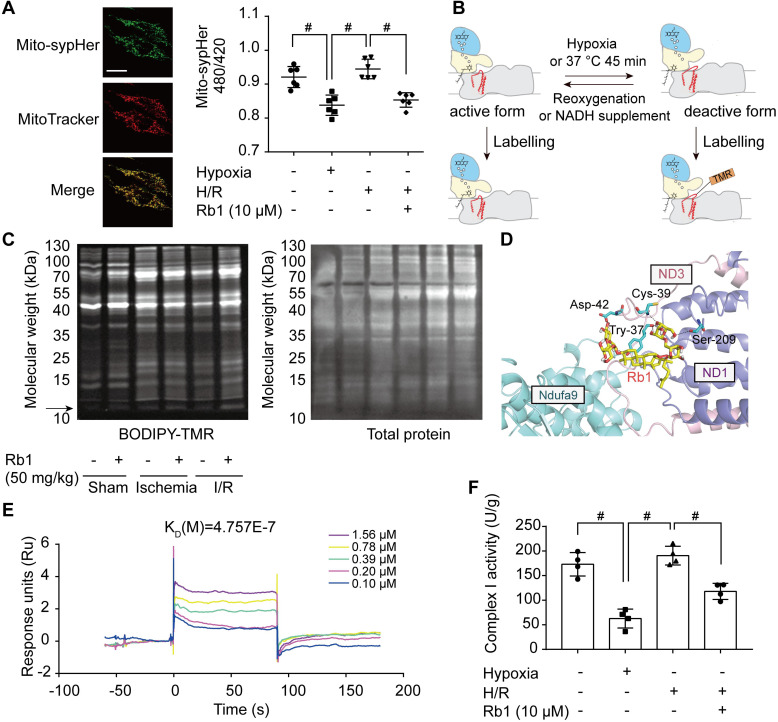
** Ginsenoside Rb1 controls the active/deactive transition of mitochondrial complex I to regulate NADH dehydrogenase activity. A**, Representative images of the mitochondrial location of mito-SypHer. Scale bar, 20 µm. Mitochondrial pH (480/420 ratio) in H9c2 cells stably transfected with mito-SypHer. **B**, Schematic diagram of BODIPY-TMR labeling of mitochondrial complex I. **C**, BODIPY-TMR signal (left) of mitochondrial proteins in the hearts of mice upon I/R injury (n = 4). **D**, Binding mode predicted by AutoDock between ginsenoside Rb1 and the mitochondrial complex I subunits involved in the active/deactive transition (ND3, ND1, and Ndufa9). **E**, SPR analysis of ginsenoside Rb1 binding to human recombinant ND3 protein. **F**, Mitochondrial complex I activity in H9c2 cells treated with ginsenoside Rb1 (10 µM) and subjected to hypoxia for 1 h with and without subsequent reoxygenation for 15 min. Data above were expressed as mean ± SD.^ #^*p* < 0.05: *vs.* indicated group. H/R, hypoxia/reoxygenation; I/R, ischemia/reperfusion; K_D_, equilibrium dissociation constant; Rb1, ginsenoside Rb1.

In I/R injury, active/deactive transitions of mitochondrial complex I are highly relevant to ROS production. The deactive form of respiratory complex I is proposed to be a Na^+^/H^+^ antiporter 34; thus, we measured mitochondrial matrix pH in H9c2 cells transfected with mito-SypHer, a radiometric mitochondrial pH indicator co-localized with MitoTracker (Figure 7A). Intramitochondrial pH was low in the ischemic state but was elevated in response to reperfusion (Figure 7A). Ginsenoside Rb1 treatment kept intramitochondrial pH at a low level upon reperfusion, suggesting that ginsenoside Rb1 maintained mitochondrial complex I in a deactive state (Figure 7A). Conformational change in response to ischemia or temperature treatment results in the exposure of critical Cys-39 within the mitochondrial subunit ND3 35, which can be labeled with thiol-specific reagents as a marker for deactivation of mitochondrial complex I (Figure 7B). Ginsenoside Rb1 treatment sustained an apparent fluorescent signal of a protein band around 10 kDa, which was then identified as a peptide of ND3 by LC-MS/MS under reactive conditions (Figure S7A, Figure S8). In addition, ginsenoside Rb1 administration prior to reperfusion preserved the exposure of ND3 in the heart of mice, further confirming the effect of ginsenoside Rb1 on mitochondrial complex I state *in vivo* (Figure 7C). To reveal the underlying mechanism, molecular docking was performed to test the potential interaction between ginsenoside Rb1 and the subunits involved in the active/deactive transition (ND3, ND1 and Ndufa9) in complex I 36. Ginsenoside Rb1 was found to interact with amino acid residues of ND3 at Cys-39, Tyr-37, and Asp-42 with a docking score of -4.73 kcal/mol (Figure 7D). To confirm if ginsenoside Rb1 directly targets ND3, the surface plasmon resonance technology was performed. Ginsenoside Rb1 exhibited a strong binding affinity for ND3 with an estimated K_D_ of 0.4757 μM (Figure 7E). These results suggested that ginsenoside Rb1 probably trapped the enzyme in a deactive conformation by binding to the ND3 subunit in complex I. The deactive state is characterized by lower NADH dehydrogenase activity compared to the active conformation 18. By limiting mitochondrial complex I in the deactive state, ginsenoside Rb1 treatment rendered a lower NADH dehydrogenase activity during reperfusion or reactivation stage (Figure 7F, Figure S7B).

## Discussion

Succinate dehydrogenase activation upon reperfusion drives ROS generation at complex I due to RET, whereas ROS production in this way can be halted by inhibiting complex I. The function of mitochondrial complex I can be influenced at different sites in different forms. Rotenone inhibits complex I activity by regulating ubiquinone to attenuate electron flux 32. Blockade of complex I activity near or at the iron-sulfur cluster contributes to the protection against ischemic damage 37. In addition, S-nitrosation of mitochondrial complex I at specific sites could slow the reactivation and limit ROS production upon reperfusion 38. Herein, we identified subunit proteins of NADH dehydrogenase in mitochondrial complex I as the potential targets regulated by ginsenoside Rb1. Because mitochondrial ROS production is an initial cause of reperfusion injury, ginsenoside Rb1 inhibited NADH dehydrogenase activity and consequently blocked ROS generation, indicative of its special role in cardioprotection.

In managing acute myocardial ischemia, timely reperfusion is essential to salvage a viable myocardium. However, this intervention is challenged by reperfusion injury. It has been reported that lethal myocardial reperfusion injury may account for up to 50% of the final infarct size 2. Considerable efforts have been devoted to alleviating reperfusion injury and improving long-term outcomes after infarction from different aspects 39-41. Although many pathophysiological factors are involved, mitochondrial ROS production has emerged as the initial cause of a cascade of events that result in tissue damage during I/R insult 6. Accompanied by impaired redox and iron homeostasis, mitochondrial dysfunction is responsible for cardiomyocyte injury 2, 7. Ginsenoside Rb1 has been demonstrated to protect the heart from I/R injury 19, and we further confirmed that this cardioprotection is largely dependent on suppression of ROS production upon reperfusion. In the ischemic heart, excessive ROS are rapidly produced at the early phase of reperfusion. Administration of ginsenoside Rb1 prior to I/R insult reduced ROS production and prevented oxidative DNA damage in the heart. Since prophylactic treatment of ischemia is not clinically feasible, we investigated ginsenoside Rb1 treatment at the onset of reperfusion and confirmed its protective effect. However, it failed to protect the heart when administered 15 min post-reperfusion. Importantly, ginsenoside Rb1 treatment prior to reperfusion afforded long-term cardiac protection. Because excessive ROS production during the first 10-20 min of reperfusion is critical for both acute and long-term heart damage 10, we reasoned that suppression of ROS burst in the early phase of reperfusion was the main protective mechanism of ginsenoside Rb1 against I/R injury. The time-specificity of any therapeutic intervention is therefore crucial to limiting ischemic heart injury.

Mitochondrial ROS is generally considered to be a random consequence of damaged ETC, since increased electron leakage generates superoxide at complexes I and III 11. Inhibition of complex I blocks the flow of electrons along the ETC, resulting in increased production of ROS 11. This occurs during conventional forward electron transport. When electrons cannot be transferred to CoQ, highly reduced FMN drives electron leakage to generate ROS. This is the mechanism by which rotenone inhibits mitochondrial complex I by binding to the CoQ site to induce ROS production 13. However, a recent study revealed that inhibition of mitochondrial complex I contributes to attenuating RET-mediated ROS burst in the early phase of reperfusion 10. During ischemia, metabolic changes reroute electrons to succinate, which accumulates and acts as an electron store. Upon reperfusion, succinate oxidation at mitochondrial complex II fuels a huge pool of electrons, leading to maximal Δψm and a highly reduced CoQ pool. Then, the high membrane potential forces electrons backward from the CoQ pool onto the FMN in complex I to generate ROS 10. Under such a condition, complex I inhibitor rotenone, by blocking reverse electron transfer, protects the heart from I/R injury 12. In the present study, ginsenoside Rb1 was found to regulate mitochondrial ETC function. It had no influence on complex IV-dependent respiration and activity, indicating that it acted on respiration upstream of complex IV. It failed to alter O_2_ consumption driven by substrates specific for complex II, further suggesting that its inhibition of RET-mediated ROS production was not due to the decrease in succinate accumulation and oxidation. Many omics technologies, such as proteomics and metabolomics, have been employed to identify potential therapeutic targets for ischemic heart diseases 42, 43. In the present study, proteomics results implied that ginsenoside Rb1 probably protected the heart from I/R injury by regulating complex I activity. It specifically inhibited complex I-dependent O_2_ consumption and enzyme activity. Together, these data indicated that ginsenoside Rb1 prevented RET-mediated ROS-induced damage by regulating complex I activity.

Complex I is a NADH:ubiquinone oxidoreductase that uses NADH oxidation and ubiquinone reduction to build the proton motive force for ATP synthesis. Mammalian complex I comprises three structural modules: N, Q, and P 44. Among them, the N-module (dehydrogenase module) contains Ndufv1, Ndufv2, Ndufs1, Ndufs4, Ndufs6, and other subunits that are responsible for oxidation of NADH and binding to FMN 45. By comparative proteomic analysis, we identified subunits of NADH dehydrogenase in mitochondrial complex I as potential effector proteins regulated by ginsenoside Rb1 when the heart was subjected to I/R insult. These proteins are complex I catalytic subunits and assembly factors that facilitate forward and reverse electron flow. Ginsenoside Rb1 downregulated these subunits, which explains why it selectively inhibited complex I-dependent mitochondrial O_2_ consumption and complex I activity rather than the activities of complexes II or IV. Consistent with published studies showing that chemical modification or genetic loss of these subunits could protect the heart from ischemic stress 46-48, we demonstrated that knockdown of these subunits reduced ROS generation and protected cardiomyocytes. To further confirm the special role of ginsenoside Rb1 in NADH dehydrogenase inhibition, we used Ndi1 as an alternative NADH dehydrogenase for overexpression in cardiomyocytes. Ndi1 is a gene encoding rotenone-insensitive internal NADH-quinone oxidoreductase of *Saccharomyces cerevisiae* mitochondria, and it can be transfected into mitochondria of mammalian cells as a replacement molecule for complex I to transfer electrons from NADH to the ubiquinone pool 33. Ginsenoside Rb1 failed to inhibit complex I activity and reduce ROS production in Ndi1-expressing cells, providing evidence to support our speculation that it prevented the burst of ROS upon reperfusion by inactivating NADH dehydrogenase. Rotenone reduces RET-driven ROS production by binding to CoQ to block reverse electron transfer, while our work further suggested NADH dehydrogenase is a potential effector that can be regulated to prevent RET-driven ROS production.

During prolonged ischemia, complex I shifts to a deactive state due to limited oxygen. Compared to the active form, the deactive conformation of complex I exhibits a lower catalytic activity, losing the ability to catalyze RET 18, 49. Upon reperfusion, complex I is rapidly reactivated for electron transfer, leading to excessive ROS generation. Theoretically, transiently locking complex I in the deactive form serves as a protective mechanism to limit ROS generation. In support of this theory, metformin has been proposed to protect the heart from ischemic injury by keeping complex I in a deactivated state 50. Exposure of Cys-39 located in the hydrophilic loop of the ND3 subunit is the molecular feature of the deactive form of complex I 35. Reversible post-translational modification of Cys-39 is a therapeutic intervention to trap complex I in the deactive form 38, 51. Similarly, compounds or pharmacological agents with the ability to target Cys-39 of mitochondrial complex I may be promising for widening the time window of intervention for I/R injury 52. In the present study, ginsenoside Rb1 sustained the exposure of ND3 subunit labeled with thiol-specific reagents at the early phase of reperfusion, suggesting its potential ability to lock complex I in a state of low activity. Further molecular docking analysis showed that ginsenoside Rb1 interacted with the hydrophilic loop of ND3 by hydrogen bonding at three amino acid residues including Cys-39. SPR analysis further suggested that ginsenoside Rb1 could bind to ND3 subunit with a high affinity. These results suggested that ginsenoside Rb1 probably trapped mitochondrial complex I in its deactive conformation by binding to the ND3 subunit. Similarly, metformin traps the enzyme in a deactive-like open-loop conformation by binding in the region of the critical ND3 loop 50. However, further studies are required to clarify the specific molecular mechanism by which ginsenoside Rb1 acts on mitochondrial complex I. Still, our results suggested that the potential mechanism for ginsenoside Rb1 action on NADH dehydrogenase activity may be derived from its regulation of the active/deactive transition of complex I. In contrast to rotenone, a tight-binding and irreversible inhibitor, ginsenoside Rb1 reversibly inhibited mitochondrial complex I activity, probably by decreasing NADH dehydrogenase activity.

Mammalian adult cardiomyocytes are terminally differentiated; therefore, the heart during adulthood is incapable of regenerating following cardiomyocyte loss, which is mostly attributable to postnatal cardiomyocyte cell cycle arrest. Therefore, the decreased infarct size observed in ginsenoside Rb1-treated mice after ischemia should be a lasting result from its initial protection against reperfusion injury. After myocardial infarction, the remodeling process initially confers protection to the heart as a compensatory regulation. However, uncontrolled fibrotic response can impair heart function due to myocardium stiffness. Because the infarcted heart is fibrotic, we examined the long-term effect of ginsenoside Rb1 after ischemia and confirmed its inhibitory effect on cardiac fibrosis. These events indicate that limiting reperfusion injury is important for functional improvement during the recovery phase. It should be noted that the improved heart function observed during the recovery phase is a final result associated with regulation from various aspects. Therefore, we cannot say with certainty that combating reperfusion injury was the only reason for the observed improvement. A comprehensive study considering mitochondrial function is necessary for full evaluation of the potential implications. Although ischemia only provides a narrow time window to reduce cardiac damage, our work emphasizes that timely therapeutic intervention is crucial for improving prognosis.

In summary, ginsenoside Rb1 modestly and reversibly inhibited mitochondrial complex I by reducing NADH dehydrogenase activity and locking mitochondrial complex I in deactive state, contributing to attenuation of myocardial reperfusion injury. These findings not only provided novel insights into the mechanism by which ginsenoside Rb1 protected the heart from ischemic injury, but also suggested that regulation of NADH dehydrogenase activity in complex I could be a potential intervention to limit reperfusion-induced mitochondrial ROS production.

## Supplementary Material

Supplementary figures and tables.

Supplementary table S3.

## Figures and Tables

**Table 1:** Mitochondrial complex I proteins regulated by ginsenoside Rb1 during the early reperfusion stage

Protein ID	Description	Abbreviation	MP	Ratio				*p-*value		
				I/R *vs*. S	Rb1 *vs*. S	Rb1 *vs.* I/R	(Rb1 *vs.* S)- (I/R *vs.* S)	I/R *vs*. S	Rb1 *vs*. S	Rb1 *vs*. I/R
Q91VD9	NADH-ubiquinone oxidoreductase 75 kDa subunit	Ndufs1	29	1.093	0.965	0.883	-0.128	0.002	0.289	0.011
Q91WD5	NADH dehydrogenase [ubiquinone] iron-sulfur protein 2	Ndufs2	15	1.122	0.978	0.872	-0.144	0.008	0.432	0.005
Q9CXZ1	NADH dehydrogenase [ubiquinone] iron-sulfur protein 4	Ndufs4	7	1.131	0.993	0.878	-0.138	0.006	0.799	0.002
Q9DC70	NADH dehydrogenase [ubiquinone] iron-sulfur protein 7	Ndufs7	7	1.087	0.999	0.919	-0.088	0.039	0.965	0.049
P52503	NADH dehydrogenase [ubiquinone] iron-sulfur protein 6	Ndufs6	6	1.074	0.896	0.834	-0.178	0.022	0.005	0.000
Q91YT0	NADH dehydrogenase [ubiquinone] flavoprotein 1	Ndufv1	18	1.090	0.967	0.887	-0.123	0.009	0.336	0.012
Q9D6J6	NADH dehydrogenase [ubiquinone] flavoprotein 2	Ndufv2	11	1.075	0.949	0.883	-0.126	0.010	0.269	0.043
Q9CQ54	NADH dehydrogenase [ubiquinone] 1 subunit C2	Ndufc2	8	1.079	1.004	0.931	-0.075	0.020	0.843	0.014
Q9CR61	NADH dehydrogenase [ubiquinone] 1 beta subcomplex subunit 7	Ndufb7	9	1.113	0.909	0.817	-0.204	0.023	0.033	0.004
Q9CQJ8	NADH dehydrogenase [ubiquinone] 1 beta subcomplex subunit 9	Ndufb9	9	1.104	0.979	0.886	-0.125	0.019	0.437	0.005
Q3UIU2	NADH dehydrogenase [ubiquinone] 1 beta subcomplex subunit 6	Ndufb6	7	1.096	0.956	0.872	-0.140	0.030	0.333	0.024
Q9D6J5	NADH dehydrogenase [ubiquinone] 1 beta subcomplex subunit 8	Ndufb8	5	1.125	0.981	0.873	-0.144	0.035	0.582	0.025
Q9CQH3	NADH dehydrogenase [ubiquinone] 1 beta subcomplex subunit 5	Ndufb5	5	1.073	0.978	0.911	-0.095	0.007	0.569	0.047
Q9CQC7	NADH dehydrogenase [ubiquinone] 1 beta subcomplex subunit 4	Ndufb4	5	1.114	1.024	0.919	-0.090	0.033	0.567	0.041
O09111	NADH dehydrogenase [ubiquinone] 1 beta subcomplex subunit 11	Ndufb11	3	1.101	1.008	0.916	-0.093	0.026	0.856	0.048
Q99LC3	NADH dehydrogenase [ubiquinone] 1 alpha subcomplex subunit 10	Ndufa10	17	1.086	0.964	0.888	-0.122	0.004	0.090	0.169
Q7TMF3	NADH dehydrogenase [ubiquinone] 1 alpha subcomplex subunit 12	Ndufa12	14	1.103	1.024	0.928	-0.079	0.048	0.598	0.002
Q9ERS2	NADH dehydrogenase [ubiquinone] 1 alpha subcomplex subunit 13	Ndufa13	9	1.088	0.982	0.903	-0.106	0.004	0.514	0.020
Q9CPP6	NADH dehydrogenase [ubiquinone] 1 alpha subcomplex subunit 5	Ndufa5	7	1.093	1.033	0.945	-0.060	0.018	0.287	0.030
Q9CQ75	NADH dehydrogenase [ubiquinone] 1 alpha subcomplex subunit 2	Ndufa2	6	1.096	0.990	0.903	-0.106	0.045	0.805	0.010

MP, matched peptides; S, sham group; I/R, ischemia/reperfusion group; Rb1, I/R plus ginsenoside Rb1 treated group.

## Abbreviations

CoQ: coenzyme Q
Δψm: mitochondrial membrane potential
ETC: electron transport chain
FMN: flavin mononucleotide
GO: gene ontology
H/R: hypoxia/reoxygenation
I/R: ischemia/reperfusion
K_D_: equilibrium dissociation constant
MPTP: mitochondrial permeability transition pore
OCR: oxygen consumption rates
RET: reverse electron transfer
ROS: reactive oxygen species
SPR: surface plasmon resonance
TMRE: tetramethylrhodamine ethyl ester
TTC: 2,3,5-triphenyltetrazolium chloride
