# Supplementary material for: Proteomic analysis reveals ginsenoside Rb1 attenuates myocardial ischemia/reperfusion injury through inhibiting ROS production from mitochondrial complex I
Source: Theranostics. 2021 Jan 1;11(4):1703–20. doi: 10.7150/thno.43895 (PMC7778584; doi:10.7150/thno.43895)

Supplementary Materials

Proteomic analysis reveals ginsenoside Rb1 attenuates myocardial ischemia/reperfusion injury through inhibiting ROS production from mitochondrial complex I

Lujing Jiang[1#], Xiaojian Yin[1#], Ya-Hui Chen[1], Yan Chen[1], Wei Jiang[1], Hao Zheng[1], Feng-Qing Huang[1,2], Baolin Liu[1], Wei Zhou[1], Lian-Wen Qi[1, 2]*, Jia Li[1]*

[1]State Key Laboratory of Natural Medicines, School of Traditional Chinese Pharmacy, China Pharmaceutical University, Nanjing 210009, Jiangsu, China.

[2]Clinical Metabolomics Center, China Pharmaceutical University, Nanjing 211198, Jiangsu, China.

***Corresponding authors:**

Jia Li, No. 639 Longmian Road, Nanjing 211198, China Pharmaceutical University, E-mail address: lijia@cpu.edu.cn

Lian-Wen Qi, No. 639 Longmian Road, Nanjing 211198, China Pharmaceutical University, E-mail address: Qilw@cpu.edu.cn

[#] The authors contributed equally to this work.

Supplementary Materials Tables

Table S1. Primer sequences used for RT-PCR.

Species	Gene	Primer Sequence 5'-3'	
Mouse	Ndufv1	Forward:	TCAGCAGGATCTCCTTTGTCT
		Reverse:	AAAACCTCATTTGGCTCACTG
Mouse	Ndufv2	Forward:	AAGGGTCTCCAATATGCTGTC
		Reverse:	ACAAGGTGGCTGAAGTTTTAC
Mouse	Ndufs1	Forward:	TGTTCCCAAATCATCTACTCC
		Reverse:	TTCTTATTAGCAAATCACCCA
Mouse	Ndufs4	Forward:	CCACATCATAGCTCCATCCGT
		Reverse:	GCTCGCAATAACATGCAGTCT
Mouse	Ndufs6	Forward:	CTAGTGATGGTGCTGCTTGAA
		Reverse:	TGAGAACTTTGCCATTGATTT
Mouse	Ndufa12	Forward:	GGATTAGTCGTCGGAGGGTCG
		Reverse:	TATAAGGATTGGTACACTGGTGGGA
Mouse	β-actin	Forward:	GGGAAATCGTGCGTGAC
		Reverse:	AGGCTGGAAAAGAGCCT

Table S2. The siRNA sequences for cell transfection.

Species	Gene	Sequence 5'-3'	
Rat	Ndufv1	sense:	GCGUUGAUUGGAUGAACAATT
		antisense:	UUGUUCAUCCAAUCAACGCTT
Rat	Ndufv2	sense:	CCGGAGGCCUUACUUCUUUTT
		antisense:	AAAGAAGUAAGGCCUCCGGTT
Rat	Ndufs1	sense:	GCUCUGAAAGAUUUGCUUATT
		antisense:	UAAGCAAAUCUUUCAGAGCTT
Rat	Ndufs4	sense:	CCAGAAAGGUCAGAAUCUUTT
		antisense:	AAGAUUCUGACCUUUCUGGTT
Rat	Ndufs6	sense:	GCCAUUGAUUUGAUAGCACTT
		antisense:	GUGCUAUCAAAUCAAUGGCTT
Rat	Ndufa12	sense:	GACUGCUCGUAAGUUCAUUTT
		antisense:	AAUGAACUUACGAGCAGUCTT

Table S3. Subcellular location of identified proteins. (Uploaded as a separate Excel file).

Supplementary Figures

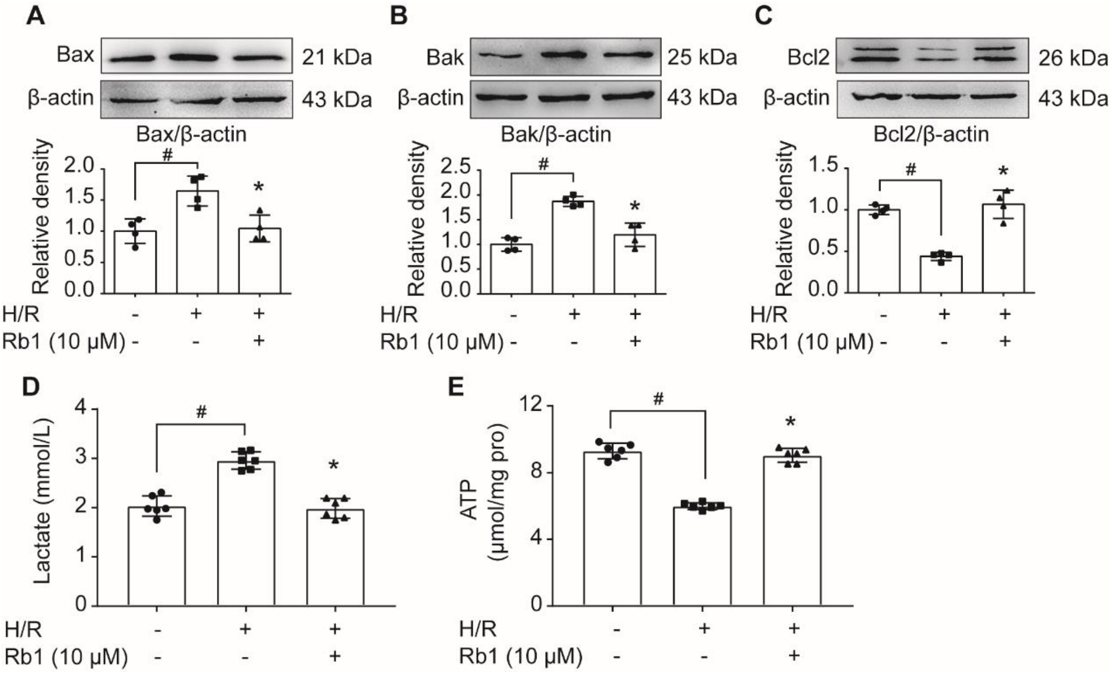

Figure S1. The effects of ginsenoside Rb1 on mitochondria-associated apoptosis proteins expressions, lactate levels, and ATP contents in response to hypoxia/reoxygenation insult. A-C, protein levels of Bax, Bak, and Bcl2 were determined by Western blot (n = 4). Lactate release (**D**, n = 6) and ATP contents (**E**, n = 6) were assayed. Data were expressed as mean ± SD. $^{*}p < 0.05$: *vs.* H/R only treatment; $^{\#}p < 0.05$: *vs.* indicated group. H/R, hypoxia/reoxygenation; Rb1, ginsenoside Rb1.

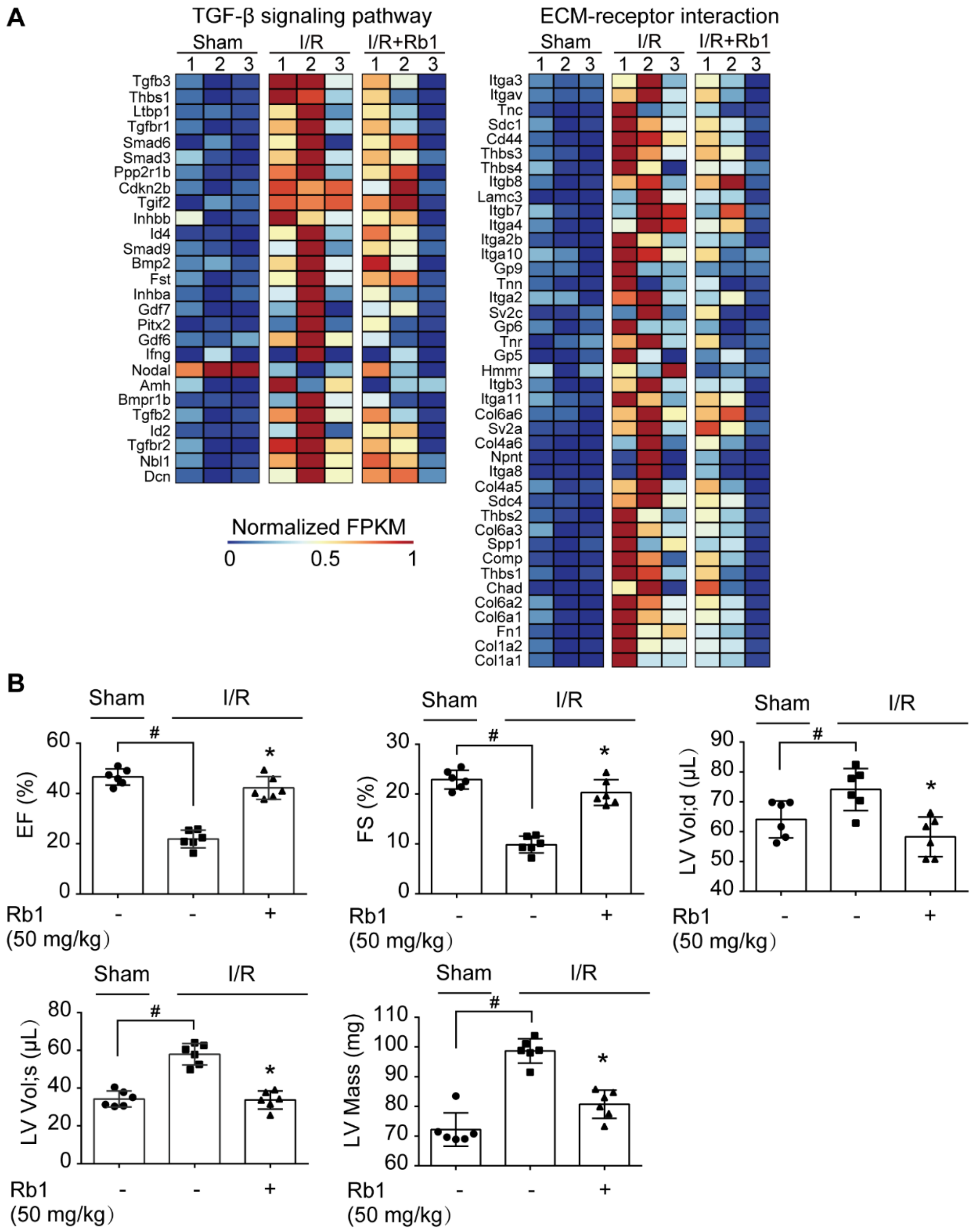

Figure S2. Heatmaps of TGF-β signaling pathway and ECM-receptor interaction (A). Heart function measured 28 days post-I/R (B, n = 6). Data were expressed as mean ± SD. $^{*}p < 0.05$: *vs.* I/R only treatment; $^{\#}p < 0.05$: *vs.* indicated group. I/R, ischemia/reperfusion. EF: ejection fraction; FS: fractional shortening; LV vol;d: left ventricular end-diastolic volume; LV vol;s: left ventricular end-systolic volume; Rb1, ginsenoside Rb1.

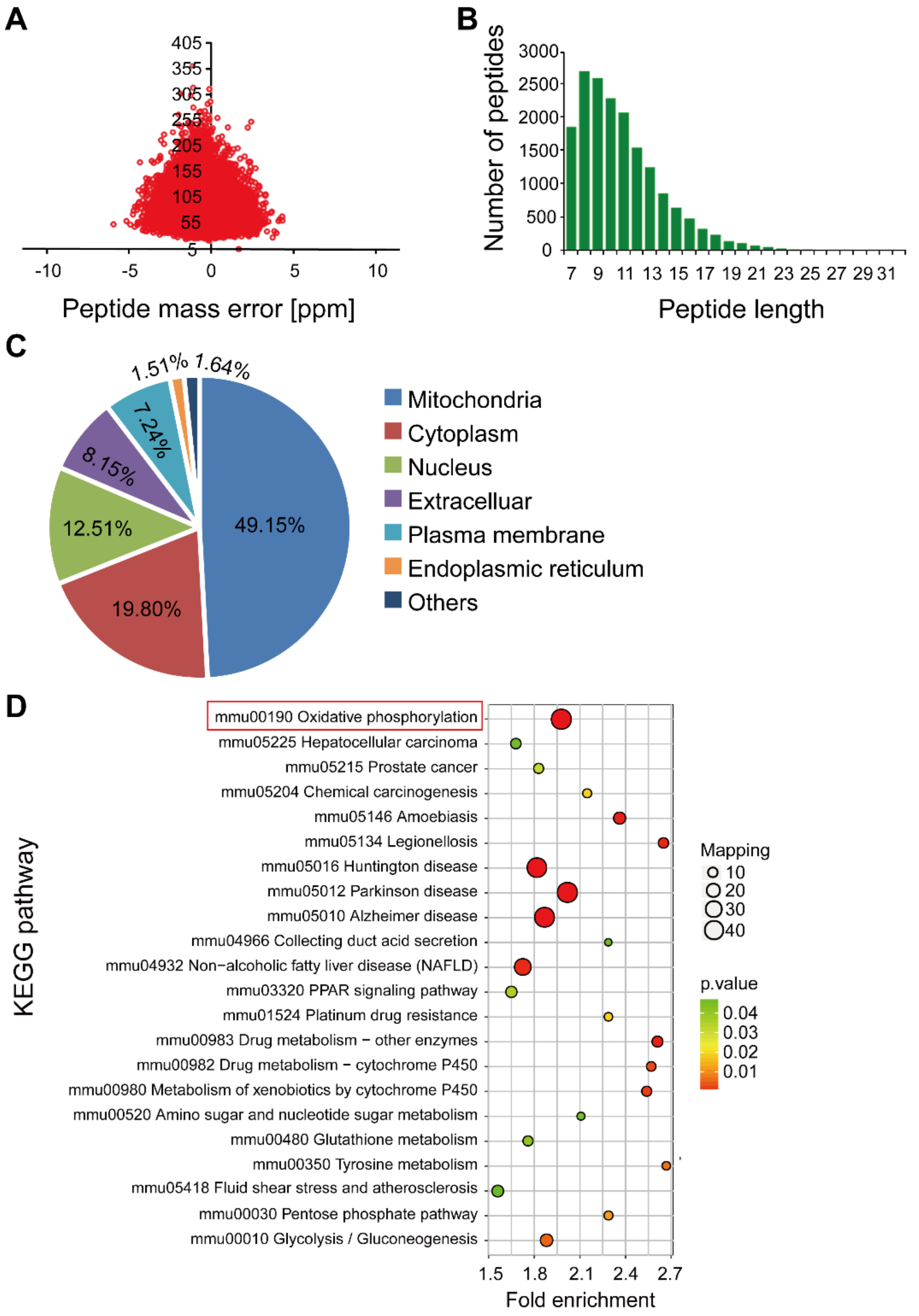

Figure S3. Quality check of proteomic data and bioinformatic analyses of significantly changed proteins. A, The mass error of identified peptides in our study. **B**, The distribution of peptides length among the identified peptides. **C**, Subcellular localization prediction of identified proteins using WoLFPSORT. The subcellular localization of identified proteins was predicated using WoLFPSORT database (https://www.genscript.com/wolf-psort.html) with amino acid sequences of identified proteins. **D**, KEGG enrichment pathway analysis.

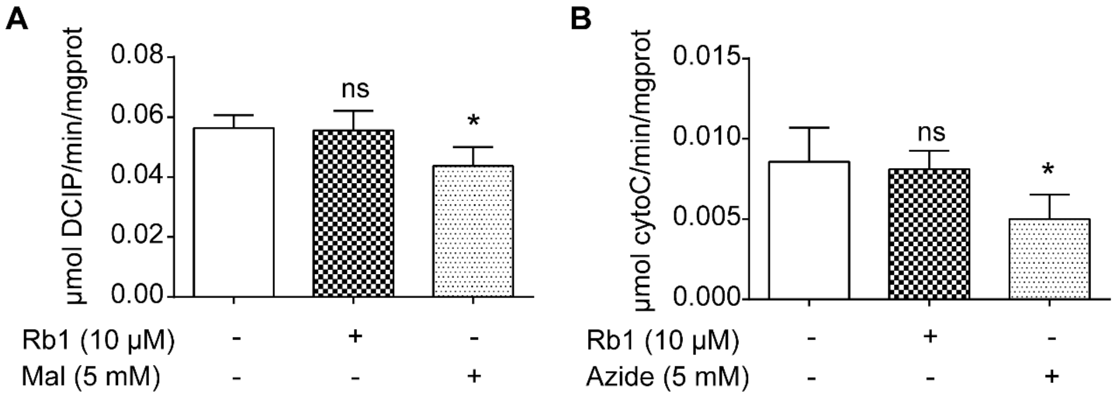

Figure S4. The effects of ginsenoside Rb1 on mitochondrial complex II and IV activities. **A**, Complex II activity. **B**, Complex IV activity. Data were expressed as mean ± SD (n = 3). $^{*}p < 0.05$: *vs* untreated control; ns. no significance *vs.* untreated control. Mal, malonate; Rb1, ginsenoside Rb1.

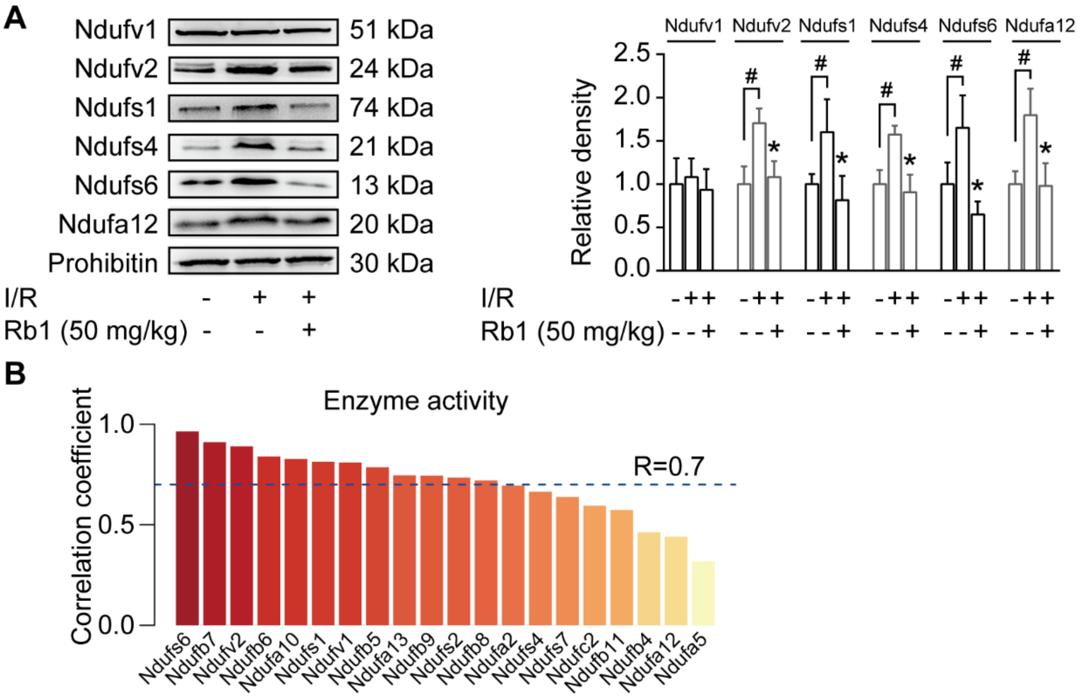

Figure S5. Validation of differentially expressed proteins by Western blot and correlation coefficients analysis. A, protein expressions of Ndufv1, Ndufv2, Ndufs1, Ndufs4, Ndufs6 and Ndufa12 in mitochondrial fraction from the hearts of mice subjected to I/R insult. **B**, Correlation coefficients (R) between NADH dehydrogenase activity and protein levels of subunits in NADH dehydrogenase, which were the differential proteins identified by proteomics in Table 1. Data were expressed as mean ± SD (n = 4). $^{*}p < 0.05$: *vs.* I/R only treatment; $^{\#}p < 0.05$: *vs.* indicated group. Rb1, ginsenoside Rb1.

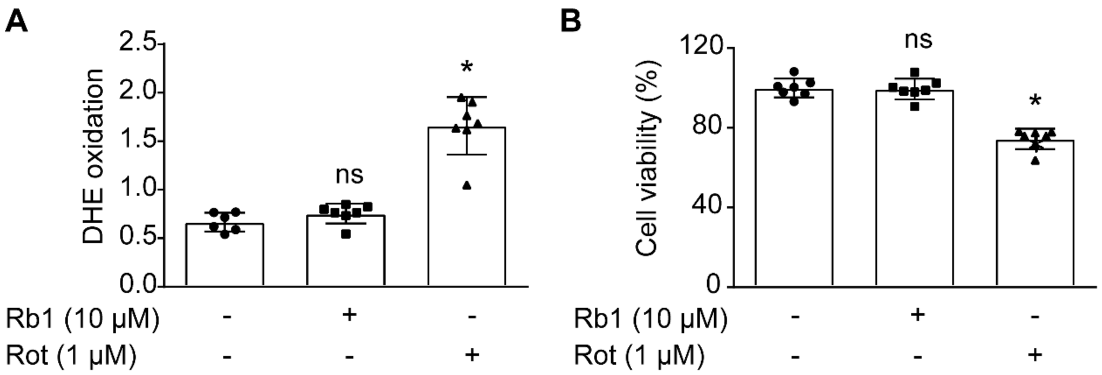

Figure S6. The effects of ginsenoside Rb1 on ROS production and cell viability under basal conditions. **A**, ROS production were measured using DHE dye. **B**, Cell viability was assayed by CCK-8 kit. Data were expressed as mean ± SD. $^{*}p < 0.05$: *vs.* untreated control. ns. no significance: *vs.* untreated control. Rb1, ginsenoside Rb1; Rot, rotenone.

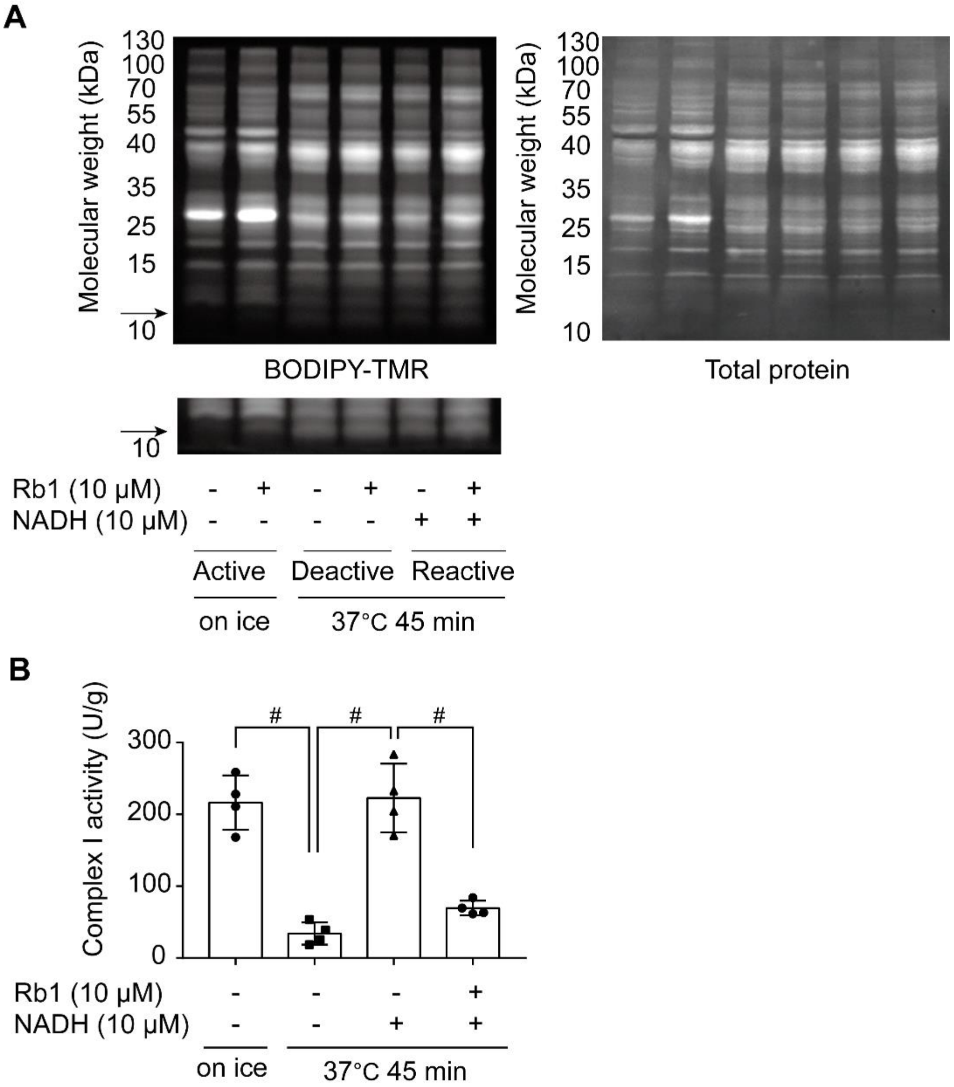

Figure S7. Ginsenoside Rb1 locked mitochondrial complex I in deactive state *in vitro*. Mitochondria were isolated from the hearts of normal mice and divided in two parts, one part was incubated at 37ºC for 45 min in the absence of substrates to deactivate complex I and the other part was kept on ice. Subsequently, the reactivation was achieved by addition of NADH. **A**, BODIPY-TMR signal (left) of mitochondrial protein. The lower image (left) is a more exposed photograph. Sypro Ruby signal (right) reflected total protein. Arrows mark the band corresponding to ND3 identified by LC-MS/MS. **B**, Mitochondrial complex I activity was measured. Data above were expressed as the mean ± SD. $^{\#}p < 0.05$: *vs.* indicated group. Rb1, ginsenoside Rb1.

Figure S8. Identification of ND3 band. (Uploaded as a separate PDF file).

Raw file	Scan	Method	Score	m/z	Gene names
LJ-kaoran-20180701	3850	ITMS; CID	15.14	549.26	Mtnd3

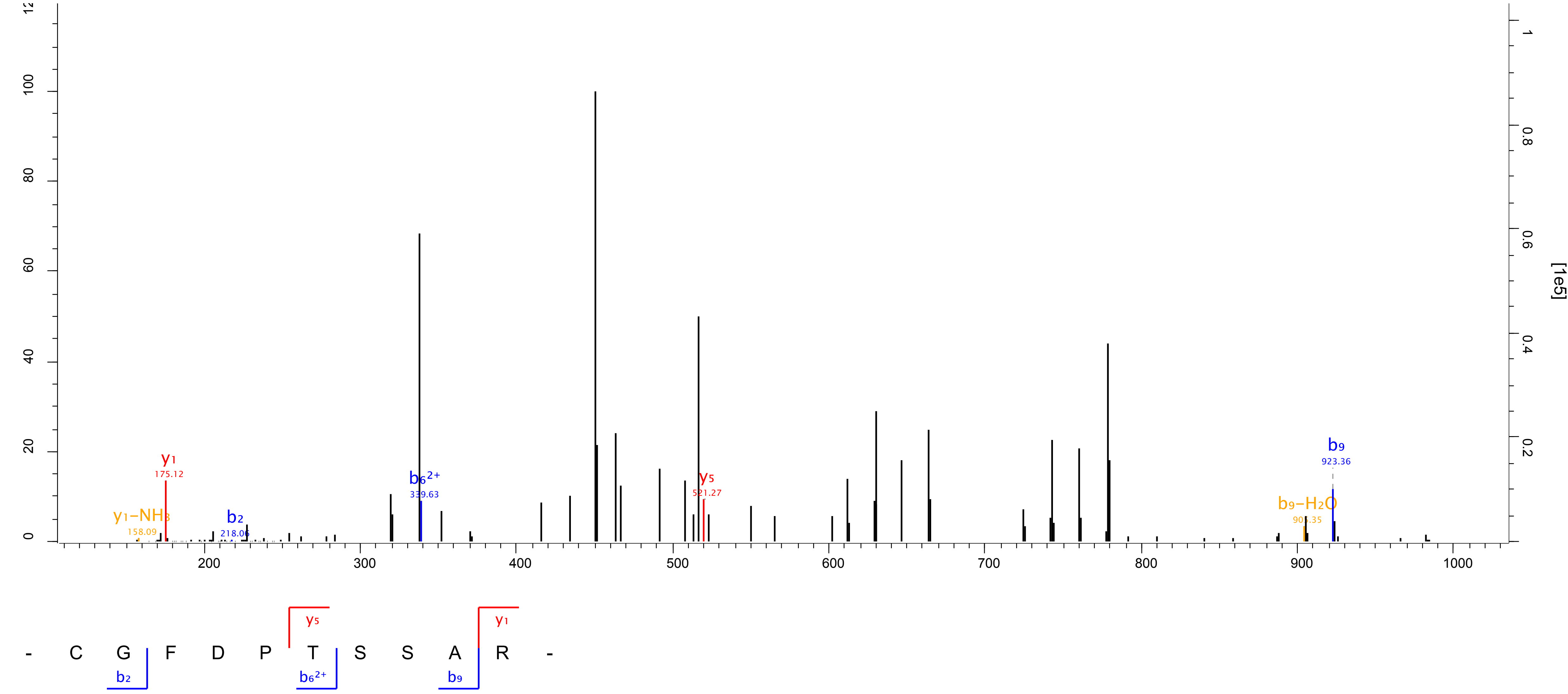

Supplement: Supplementary file 1 — Supplementary figures and tables. [file thnov11p1703s1.pdf]
